# Sex- and age-specific burden of self-harm and suicide mortality: A national and subnational study in Iran

**DOI:** 10.1017/gmh.2025.10104

**Published:** 2026-01-19

**Authors:** Sohrab Amiri, Jannat Mashayekhi

**Affiliations:** Spiritual Health Research Center, Lifestyle Institute, Baqiyatallah University of Medical Sciences, Tehran, Iran

**Keywords:** prevalence, incidence, self-harm, suicide mortality, global burden of disease

## Abstract

This study focuses on the national and subnational estimation of prevalence, incidence, disability-adjusted life years (DALYs) related to self-harm and suicide mortality in Iran. These indicators of disease burden were analyzed over the period from 1990 to 2021, with stratifications based on sex, age and geographic location. Additionally, the percentage change observed between 1990 and 2021 was documented. The age-standardized prevalence rate (per 100,000) of self-harm decreased from 173.92 (95% UI: 146.13–208.75) in 1990 to 131.2 (95% UI: 110.55–156.67) in 2021, reflecting a percentage change of −0.25% over the period. In terms of self-harm prevalence in 2021, males had a higher rate (137.62 per 100,000) compared to females (124.82 per 100,000). The findings of the current study revealed that, despite significant challenges such as demographic shifts, economic instability and the impacts of war, the trends in self-harm incidents and suicide mortality rates in Iran have generally been on the decline. Additionally, it was observed that suicide-related deaths were more prevalent among males when compared to females. However, when examining self-harm behaviors over previous decades, these acts appeared to be more frequent among females.

## Impact statement

One of the important factors in self-harm and suicide mortality is geographical differences, which lead to cultural and religious differences. Consequently, during these years, the nation grappled with a range of issues stemming from the aftermath of war, including an elevated incidence of mental health disorders, economic instability and pressures associated with international policies. Recognizing the significant role of mental health in enhancing overall community well-being, health policies should prioritize expanding access to mental health care services. This can be achieved through proactive initiatives that focus on education at various levels, including community programs, schools, universities and health centers. Efforts should aim to improve mental health literacy while addressing cases requiring psychological interventions, thereby reducing the economic and social impact of mental health issues.

## Introduction

Self-harm and suicide represent significant health and social challenges on a global scale (Knipe et al., 2022). The World Health Organization (WHO) conceptualizes self-harm as the act of intentionally causing injury to oneself as a means of managing or expressing severe emotional distress and internal conflict. While individuals engaging in such behavior typically do not aim to end their lives, the potential consequences can nonetheless be fatal. Common manifestations of self-harm include deliberate poisoning through the ingestion of an excessive amount of medication or harmful substances, self-inflicted wounds through cutting or burning, head-banging against solid objects or inflicting physical pain by punching or striking oneself against hard surfaces. Although suicidal intent is generally absent among those who engage in self-harm, the risks associated with these actions remain profoundly serious (World Health Organization, 2019). Suicide can be defined as “the act of intentionally carrying out an action to kill oneself” (World Health Organization, 2019).

The WHO estimates that over 700,000 individuals lose their lives to suicide annually worldwide (World Health Organization, 2023). In 2019, the global age-standardized suicide rate stood at 9.0 per 100,000 people, with men exhibiting a higher rate of 12.6 per 100,000 compared to women, who had a rate of 5.4 per 100,000 (World Health Organization, 2021). Although suicide mortality rates are more common in males, self-harm is more common in females (Knipe et al., 2022). Self-harm and suicide result from the interaction of complex factors and are influenced by variables such as age, sex, ethnicity and geography (Knipe et al., 2022).

The region encompassing North Africa and the Middle East, including Iran, has experienced significant challenges in recent decades, marked by rapid population growth, ongoing conflicts and persistent warfare. The nations within the Middle East exhibit substantial ethnic and demographic commonalities, as noted (Moradinazar et al., 2022). Islam serves as the predominant religion across these countries. While historically, the rate of suicide has generally been low in most Islamic societies, contemporary evidence suggests a concerning upward trajectory in this phenomenon (Pritchard and Amanullah, 2007; Mirhashemi et al., 2016). In Iran, the overall suicide rate is 5.3 per 100,000, with rates of 7 and 3.6 for males and females respectively (World Health Organization, 2014). Some determinants of suicide in Iran include family conflict, marital problems, economic constraints and educational failures (Nazarzadeh et al., 2013).

During the past decades, Iran has been affected by economic turmoil, war and international politics (Danaei et al., 2019). After the end of the war in 1988, Iran entered a period of construction in health and nonhealth infrastructures, which led to an increase in gross domestic product (Bank, 2021). Demographic transition has led to population growth, a change in fertility, increased life expectancy, aging and epidemiological transition (Farzadfar et al., 2022; Bhattacharjee et al., 2024; GBD 2021 Causes of Death Collaborators, 2024). Access to mental health care services has not been developed in response to the growth of mental disorders (Farzadfar et al., 2022). The prevalence of mental health issues has gained considerable attention since the conclusion of the Iran–Iraq war. This concern is further underscored by the findings of the Iranian Mental Health Survey, which indicates a substantial occurrence of psychiatric disorders within the country (Sharifi et al., 2015; Danaei et al., 2019). This study focuses on estimating the prevalence, incidence and disability-adjusted life years (DALYs) associated with self-harm and suicide mortality in Iran at both national and subnational levels, using data from the Global Burden of Disease (GBD) 2021.

## Methods

### Protocol

This manuscript was produced as part of the GBD Collaborator Network and following the GBD Protocol.

### Data source

This research was based on the GBD 2021 (Ferrari et al., 2024). The extracted burden of disease indicators included prevalence, incidence, DALYs, years lived with disability (YLDs), years of life lost (YLLs) and death for 371 diseases and injuries, along with estimates of healthy life expectancy. These estimates are provided for sex and age groups, and for 204 countries and territories, including subnational estimates for 21 countries (Ferrari et al., 2024). Data sources used in GBD 2021 included 100,983 data sources (19,189 new data sources for DALYs), 12 new causes and other important methodological updates (Ferrari et al., 2024). These indicators were examined at the national and subnational levels in Iran. More details of GBD 2021 are presented elsewhere (Ferrari et al., 2024).

### Case definitions

Self-harm in GBD 2021 is “deliberate bodily damage inflicted on oneself resulting in death or injury. ICD-9: E950-E959; ICD-10: X60-X64.9, X66-X84.9, Y87.0” (2021, 2024; Ferrari et al., 2024). This contains two subclasses: (i) Self-harm by firearm, defined as “Death or disability inflicted by the intentional use of a firearm on oneself. ICD-9: E955-E955.9; ICD-10: X72-X74.9” (2021, 2024; Ferrari et al., 2024); and (ii) Self-harm by other specified means, defined as “Death or occurrence of deliberate bodily damage inflicted on oneself resulting in death by means of self-poisoning, medication overdose, transport, falling from height, hanging or strangulation or other mechanisms not including firearms. ICD9: E950-E954, E956-E959; ICD10: X60-X64.9, X66-X67.9, X69-X71.9, X75-X75.9, X77-X84.9, Y87.0” (2021, 2024; Ferrari et al., 2024).

### Estimation framework

YLDs were calculated “with a microsimulation process that used estimated age-sex-location-year-specific prevalent counts of nonfatal disease sequelae (consequences of a disease or injury) for each cause and disability weights for each sequela as the input estimates at varying levels of severity by an appropriate disability weight” (Ferrari et al., 2024). YLLs were calculated as “the product of estimated age-sex-location-year-specific deaths and the standard life expectancy at the age death occurred for a given cause” (Ferrari et al., 2024). DALYs were calculated as the sum of YLDs and YLLs (Ferrari et al., 2024).

### Statistics

All-age count estimates and age-standardized rate prevalence (per 100,000) were calculated for prevalence, incidence, DALYs and death. Each of the disease burden indicators was examined in the period of 1990–2021, stratified by sex, age and location, and the % change between 1990 and 2021 was reported. The 95% uncertainty interval (UI) was reported for each of the reported estimates. More details about data, data processing and modeling are provided elsewhere and are related to GBD 2021 (Ferrari et al., 2024). GBD 2021 complies with the Guidelines for Accurate and Transparent Health Estimates Reporting (Stevens et al., 2016) and analyses were completed using Python (version 3.10.4), Stata (version 13.1) and R (version 4.2.1).

## Results

### Prevalence of self-harm in Iran from 1990 to 2021

**Figure 1.**
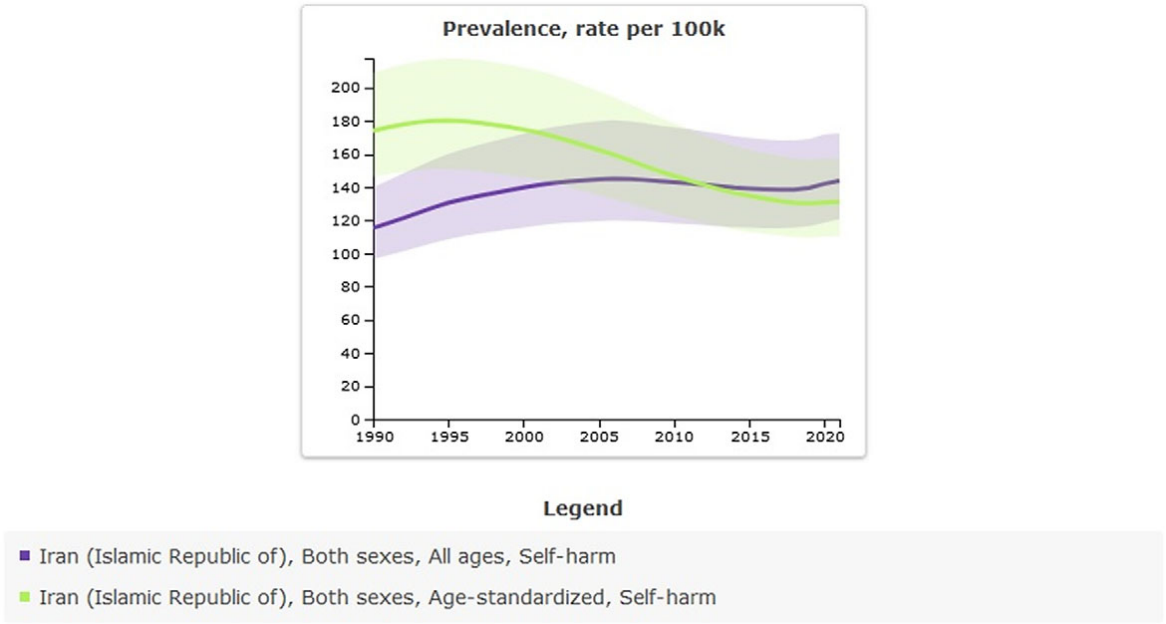
Trend in prevalence of self-harm in Iran, 1990–2021.

Age-standardized rate prevalence (per 100,000) of self-harm in 1990 was 173.92 (95% UI: 146.13–208.75) versus 131.2 (95% UI: 110.55–156.67) in 2021, thus indicating a decrease; the percentage change from 1990 to 2021 was −25%. All-ages count estimates of self-harm in 1990 were 65,834 (95% UI: 55,038–79,967) versus 122,781 (95% UI: 103,040–147,165) in 2021; the percentage change from 1990 to 2021 was 0.86%. While the age-standardized rate has shown a decline, the absolute count estimates have increased over recent decades, primarily due to population growth (Table 1 and Figure 1).

**Table 1.** All-ages counts and age-standardized rate (per 100,000) prevalence of self-harm in Iran, stratified by provinces, 1990–2021

Location	Year								
	1990			2021			Percentage change 1990–2021		
	Value	Lower	Upper	Value	Lower	Upper	Value	Lower	Upper
Age-standardized rate prevalence (per 100,000)									
Iran	173.92	146.13	208.75	131.2	110.55	156.67	−0.25	−0.28	−0.22
Alborz	178.5	149.19	214.24	152.64	127.58	181.88	−0.14	−0.17	−0.12
Ardebil	203.51	171.14	243.77	139.42	117.74	166.11	−0.31	−0.35	−0.28
Bushehr	184.88	155.03	221.06	125.95	107.24	149.5	−0.32	−0.36	−0.28
Chahar Mahaal and Bakhtiari	212.45	177.76	253.55	132.24	110.95	157.88	−0.38	−0.41	−0.35
East Azarbayejan	175.65	146.19	215.15	137.76	115.82	164.44	−0.22	−0.26	−0.18
Fars	211.5	175.97	255.93	150.55	125.4	180.46	−0.29	−0.32	−0.26
Gilan	158.59	131.6	191.18	133.92	112.27	159.34	−0.16	−0.19	−0.12
Golestan	228.88	189.83	279.77	147.88	123.62	177.4	−0.35	−0.39	−0.32
Hamadan	209.33	173.99	255.23	160.45	134.13	191	−0.23	−0.28	−0.19
Hormozgan	165.42	139.31	196.97	126.22	106.7	151.08	−0.24	−0.27	−0.21
Ilam	276.54	230.25	334.34	188.33	157.11	223.85	−0.32	−0.35	−0.29
Isfahan	133.54	112.54	160.13	117.3	98.45	140.14	−0.12	−0.15	−0.1
Kerman	165.18	137.85	199.46	131.3	110.3	157.52	−0.21	−0.24	−0.18
Kermanshah	301.39	251.47	370.07	178.76	148.3	213.86	−0.41	−0.44	−0.38
Khorasan-e-Razavi	169.05	141.02	204.91	125.02	105.77	148.83	−0.26	−0.3	−0.22
Khuzestan	185.52	155.27	222.52	140.34	117.2	169.14	−0.24	−0.27	−0.22
Kohgiluyeh and Boyer-Ahmad	226.6	188.32	278.46	169.1	140.05	203.16	−0.25	−0.29	−0.22
Kurdistan	217.73	182.21	262.47	131.14	110.26	156.09	−0.4	−0.43	−0.37
Lorestan	251.81	210.01	303.26	174.12	144.77	205.66	−0.31	−0.34	−0.27
Markazi	162.87	136.18	196.35	114.55	96.64	136.53	−0.3	−0.33	−0.26
Mazandaran	134.46	112.75	161.84	122.26	102.44	146.53	−0.09	−0.12	−0.06
North Khorasan	205.6	172.25	250.6	140.01	117.22	167.14	−0.32	−0.35	−0.29
Qazvin	122.68	103.93	146.4	116.23	98.39	138.28	−0.05	−0.09	−0.02
Qom	123.74	105.3	147.14	108.72	92.02	129.28	−0.12	−0.15	−0.09
Semnan	123.04	103.9	147.66	109.19	92.72	129.22	−0.11	−0.15	−0.08
Sistan and Baluchistan	115.68	99.28	137.06	121.66	103.55	144.79	0.05	0.02	0.08
South Khorasan	138.54	117.01	165.56	122.12	103.87	146.15	−0.12	−0.16	−0.09
Tehran	146.96	125.22	173.76	109.65	93.76	129.7	−0.25	−0.28	−0.23
West Azarbayejan	209.73	174.08	258.42	155.46	129.71	185.95	−0.26	−0.3	−0.22
Yazd	139.26	118.03	165.83	112.4	94.88	133.54	−0.19	−0.23	−0.17
Zanjan	124.69	104.09	150.7	106.84	90.84	127.12	−0.14	−0.19	−0.11
All-ages counts estimates									
Iran	65,834.87	55,038.75	79,967.61	122,781.76	103,040.92	147,165.99	0.86	0.75	0.97
Alborz	1,696.82	1,405.40	2,081.74	5,259.88	4,377.23	6,314.42	2.1	1.93	2.27
Ardebil	1,436.58	1,198.00	1,739.51	2,019.58	1,696.22	2,413.46	0.41	0.31	0.5
Bushehr	804.56	667.29	983.66	1,573.10	1,329.39	1,881.32	0.96	0.81	1.08
Chahar Mahaal and Bakhtiari	914.4	760.57	1,108.48	1,413.89	1,179.86	1,700.93	0.55	0.44	0.64
East Azarbayejan	4,103.35	3,397.43	5,076.82	6,465.85	5,421.25	7,712.64	0.58	0.45	0.69
Fars	4,935.51	4,059.97	6,077.20	8,718.66	7,230.23	10,491.51	0.77	0.65	0.88
Gilan	2,643.18	2,187.74	3,231.66	4,440.05	3,727.77	5,293.25	0.68	0.58	0.79
Golestan	1,899.27	1,564.14	2,373.57	3,029.73	2,520.14	3,653.02	0.6	0.46	0.71
Hamadan	2,341.79	1,934.59	2,891.69	3,232.73	2,694.26	3,851.66	0.38	0.27	0.48
Hormozgan	934.97	782.04	1,127.97	2,362.41	1,971.94	2,852.77	1.53	1.39	1.63
Ilam	714.73	584.95	884.35	1,267.53	1,049.95	1,523.53	0.77	0.64	0.89
Isfahan	3,447.57	2,892.63	4,196.77	7,361.04	6,172.07	8,820.49	1.14	1.01	1.25
Kerman	1,943.91	1,620.02	2,375.54	4,561.80	3,801.49	5,503.58	1.35	1.22	1.47
Kermanshah	3,285.21	2,701.59	4,109.75	4,128.72	3,416.37	4,953.89	0.26	0.16	0.34
Khorasan-e-Razavi	5,164.58	4,300.56	6,309.27	8,880.43	7,477.54	10,626.20	0.72	0.59	0.83
Khuzestan	3,535.05	2,942.60	4,346.78	7,056.76	5,847.49	8,562.09	1	0.9	1.09
Kohgiluyeh and Boyer-Ahmad	635.97	521.02	801.32	1,333.77	1,093.08	1,631.27	1.1	0.95	1.23
Kurdistan	1,726.69	1,432.93	2,120.29	2,496.20	2,088.58	2,990.30	0.45	0.34	0.54
Lorestan	2,365.37	1,961.41	2,913.33	3,347.92	2,773.13	3,989.78	0.42	0.32	0.51
Markazi	1,327.51	1,111.45	1,616.79	1,950.96	1,643.72	2,325.07	0.47	0.36	0.57
Mazandaran	2,368.55	1,972.35	2,909.53	5,241.43	4,394.54	6,269.77	1.21	1.09	1.35
North Khorasan	855.31	712.79	1,061.82	1,256.70	1,047.33	1,508.13	0.47	0.36	0.55
Qazvin	735.99	617.77	893.05	1,748.59	1,475.38	2,088.49	1.38	1.24	1.51
Qom	578.08	487.18	702.7	1,618.84	1,361.07	1,939.42	1.8	1.64	1.97
Semnan	423.16	356.45	510.08	945.74	799.38	1,128.53	1.23	1.13	1.33
Sistan and Baluchistan	967.64	822.49	1,157.74	2,888.69	2,427.41	3,464.85	1.99	1.88	2.09
South Khorasan	614.73	519.05	739.81	1,076.21	911.1	1,291.98	0.75	0.66	0.83
Tehran	8,903.83	7,516.98	10,694.68	18,736.82	15,979.39	22,234.83	1.1	0.99	1.2
West Azarbayejan	3,193.78	2,621.41	4,007.53	5,687.11	4,723.61	6,828.00	0.78	0.65	0.9
Yazd	635.95	536.06	769.85	1,381.60	1,160.92	1,651.76	1.17	1.06	1.28
Zanjan	700.84	583.61	860.52	1,299.00	1,099.52	1,550.85	0.85	0.74	0.96

### Incidence of self-harm in Iran from 1990 to 2021

**Figure 2.**
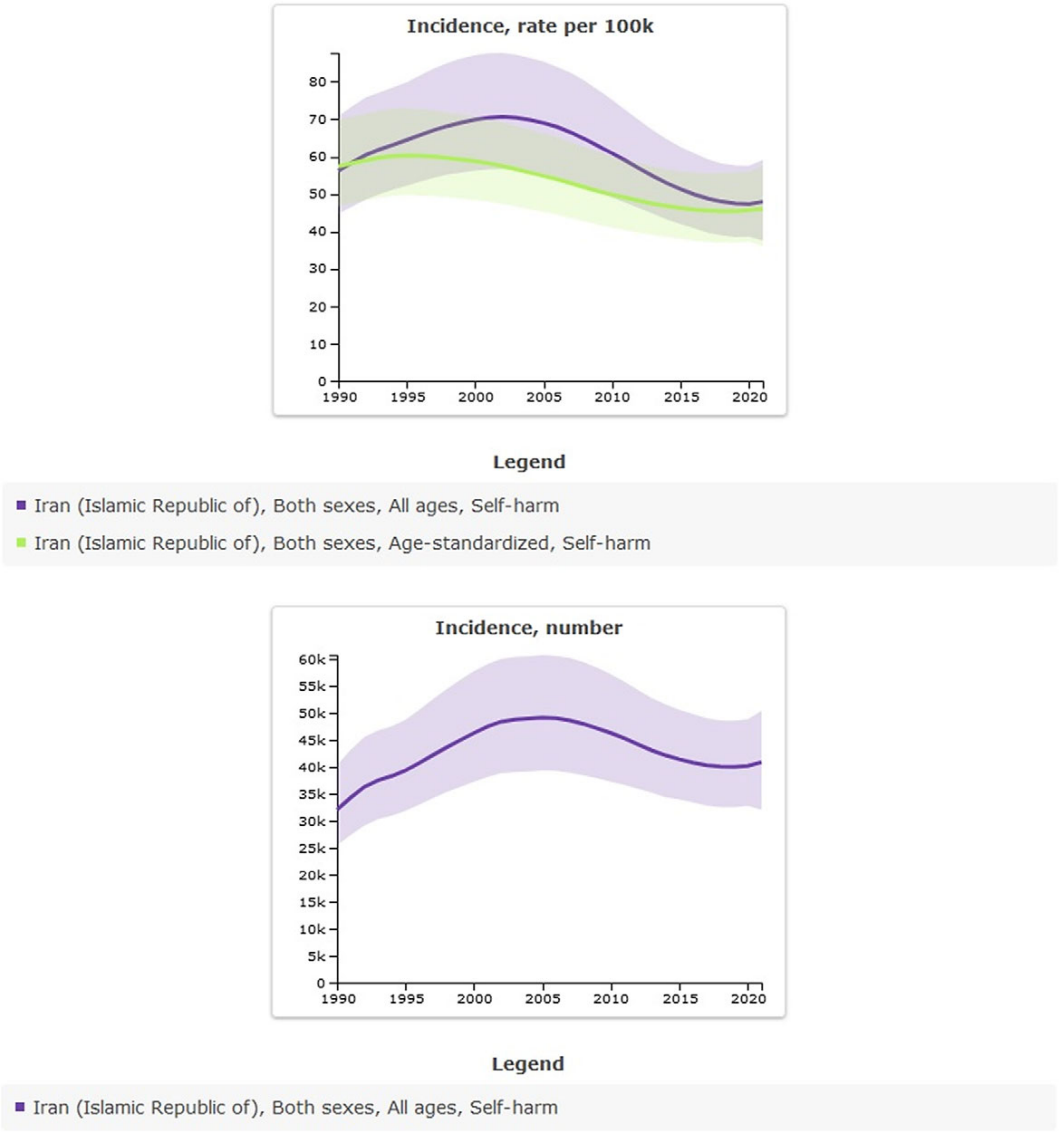
Trend in incidence of self-harm in Iran, 1990–2021.

The age-standardized incidence rate of self-harm per 100,000 population was 57.34 (95% UI: 46.67–69.95) in 1990, compared to 46.01 (95% UI: 36.07–57.53) in 2021. This reflects a percentage change of −0.20% over the period, indicating a downward trend. All-ages count estimates increased from 32,083.13 (95% UI: 25,531–40,402) in 1990 to 40,908.57 (95% UI: 32,066.60–50,434.56) in 2021; the percentage change from 1990 to 2021 was 0.28%. Between 2000 and 2010, the estimated incidence of self-harm showed an overall increase, peaking in 2005. Nevertheless, when adjusted for age, the incidence rates indicate a declining trend (Table 2 and Figure 2).

**Table 2.** All-ages counts and age-standardized rate (per 100,000) incidence of self-harm in Iran, stratified by provinces, 1990–2021

Location	Year								
	1990			2021			Percentage change 1990–2021		
	Value	Lower	Upper	Value	Lower	Upper	Value	Lower	Upper
Age-standardized rate incidence (per 100,000)									
Iran	57.34	46.67	69.95	46.01	36.07	57.53	−0.2	−0.26	−0.15
Alborz	58.75	47.85	71.94	54.12	42.58	67.02	−0.08	−0.15	−0.01
Ardebil	66.26	53.35	81.24	48.96	38.34	61.13	−0.26	−0.32	−0.2
Bushehr	62.26	50.4	76.03	45.3	35.66	56.3	−0.27	−0.34	−0.21
Chahar Mahaal and Bakhtiari	71.82	58.3	88.23	48.59	38.34	61.09	−0.32	−0.38	−0.27
East Azarbayejan	55.48	44.64	68.17	47.45	36.97	59.62	−0.14	−0.22	−0.06
Fars	69.82	56.66	85.59	52.91	41.66	66.62	−0.24	−0.3	−0.18
Gilan	55.97	45.52	68.17	47.72	37.45	59.53	−0.15	−0.23	−0.08
Golestan	75.49	60.77	92.1	51.35	40.35	63.55	−0.32	−0.38	−0.26
Hamadan	66.09	53.63	80.8	55.26	42.9	68.57	−0.16	−0.24	−0.09
Hormozgan	52.5	42.54	64.54	42.75	33.5	53.47	−0.19	−0.25	−0.11
Ilam	94.06	76.65	114.12	67.71	53.48	83.73	−0.28	−0.34	−0.22
Isfahan	43.51	35.1	53.68	41	32.37	51.27	−0.06	−0.13	0.01
Kerman	52.94	42.66	65.05	44.85	34.99	56.4	−0.15	−0.23	−0.07
Kermanshah	98.99	81.37	121.18	62.73	49.26	77.69	−0.37	−0.42	−0.31
Khorasan-e-Razavi	54.11	43.74	67.21	42.95	33.59	53.64	−0.21	−0.27	−0.14
Khuzestan	61.47	49.24	75.94	49.07	38.3	61.32	−0.2	−0.26	−0.15
Kohgiluyeh and Boyer-Ahmad	75.31	60.75	92.62	60.5	47.55	76.12	−0.2	−0.26	−0.12
Kurdistan	71.16	57.59	86.6	45.7	35.79	57.71	−0.36	−0.42	−0.3
Lorestan	83.76	67.77	102.28	63.28	49.55	78.07	−0.24	−0.3	−0.19
Markazi	51.66	41.77	62.96	39.46	31.08	49.8	−0.24	−0.3	−0.17
Mazandaran	44.86	36.35	54.98	43.56	34.06	54.22	−0.03	−0.1	0.05
North Khorasan	66.26	53.58	80.45	48.4	38.25	60.81	−0.27	−0.33	−0.21
Qazvin	39.42	31.94	48.2	40.52	31.88	50.76	0.03	−0.05	0.13
Qom	38.71	31.52	47.94	37.23	29.02	46.83	−0.04	−0.12	0.05
Semnan	39.16	31.78	48.14	38.01	29.58	47.79	−0.03	−0.11	0.04
Sistan and Baluchistan	36.32	29.18	44.62	39.48	30.79	50.01	0.09	0.02	0.16
South Khorasan	44.36	36.08	54.6	42.09	33.09	53.21	−0.05	−0.13	0.03
Tehran	48.8	39.64	59.27	39.32	30.69	49.46	−0.19	−0.26	−0.14
West Azarbayejan	67.54	54.44	83.27	54.2	42.62	67.46	−0.2	−0.26	−0.13
Yazd	45.25	36.24	55.14	39.36	30.72	49.83	−0.13	−0.2	−0.06
Zanjan	42.44	34.25	51.99	37.43	29.29	46.57	−0.12	−0.19	−0.05
All-ages counts estimates									
Iran	32,083.13	25,531.07	40,402.50	40,908.57	32,066.60	50,434.56	0.28	0.13	0.45
Alborz	852.84	677.61	1,065.37	1,738.63	1,360.77	2,121.95	1.04	0.79	1.34
Ardebil	726.2	571.52	906.22	674.68	530.38	833.61	−0.07	−0.2	0.08
Bushehr	428.98	339.05	541.65	618.96	485.3	763.99	0.44	0.28	0.64
Chahar Mahaal and Bakhtiari	484.87	381.39	608.4	507.2	402.47	632.45	0.05	−0.09	0.21
East Azarbayejan	1,905.05	1,495.14	2,426.58	1,990.47	1,551.77	2,469.60	0.04	−0.1	0.21
Fars	2,468.30	1,952.91	3,084.60	2,866.83	2,245.32	3,581.91	0.16	0.02	0.34
Gilan	1,290.10	1,030.44	1,602.01	1,280.72	1,020.46	1,574.78	−0.01	−0.15	0.16
Golestan	962.84	761.62	1,221.29	1,026.12	811.59	1,266.94	0.07	−0.06	0.21
Hamadan	1,108.85	875.84	1,399.66	988.45	775.47	1,221.09	−0.11	−0.23	0.03
Hormozgan	448.97	355.86	563.39	901.75	699.31	1,116.00	1.01	0.76	1.24
Ilam	399.7	319.8	502.48	449.74	355.03	561.26	0.13	−0.04	0.32
Isfahan	1,645.50	1,299.43	2,083.79	2,271.98	1,780.54	2,781.57	0.38	0.2	0.6
Kerman	928.84	731.49	1,170.48	1,594.94	1,231.80	1,995.61	0.72	0.5	0.97
Kermanshah	1,634.78	1,304.50	2,037.65	1,315.87	1,040.43	1,633.22	−0.2	−0.3	−0.07
Khorasan-e-Razavi	2,429.10	1,920.42	3,086.05	3,057.46	2,402.54	3,775.67	0.26	0.1	0.43
Khuzestan	1,843.14	1,443.24	2,343.91	2,517.80	1,960.87	3,115.10	0.37	0.21	0.55
Kohgiluyeh and Boyer-Ahmad	357.38	282.13	458.36	506.33	392.96	638.21	0.42	0.22	0.65
Kurdistan	853.51	672.77	1,065.77	841.78	660.22	1,047.65	−0.01	−0.15	0.14
Lorestan	1,248.81	988.24	1,581.47	1,189.21	934.28	1,464.29	−0.05	−0.17	0.1
Markazi	612.27	485.14	771.58	601.49	474.71	749.9	−0.02	−0.15	0.13
Mazandaran	1,169.66	926.07	1,457.30	1,577.38	1,235.91	1,932.35	0.35	0.18	0.58
North Khorasan	406.36	319.96	508.8	429.77	339.25	534.98	0.06	−0.06	0.2
Qazvin	361.49	283.59	457.61	580.87	457.06	719.76	0.61	0.41	0.88
Qom	277.67	219.6	354.37	566.56	437.16	706	1.04	0.77	1.32
Semnan	183.86	146.88	232.81	328.01	256.54	410.98	0.78	0.56	1.03
Sistan and Baluchistan	468.26	366.97	603.82	1,248.74	958.94	1,614.82	1.67	1.45	1.92
South Khorasan	268.34	213.38	339.46	383.37	300.94	478.68	0.43	0.27	0.61
Tehran	4,091.57	3,277.09	5,103.61	5,986.92	4,666.01	7,380.59	0.46	0.3	0.66
West Azarbayejan	1,580.07	1,241.56	1,994.11	1,948.27	1,544.81	2,397.27	0.23	0.08	0.42
Yazd	291.25	229.19	363.95	488.96	380.83	613.11	0.68	0.49	0.89
Zanjan	354.59	279.96	448.1	429.31	337.33	532.67	0.21	0.04	0.41

### DALYs of self-harm in Iran from 1990 to 2021

**Figure 3.**
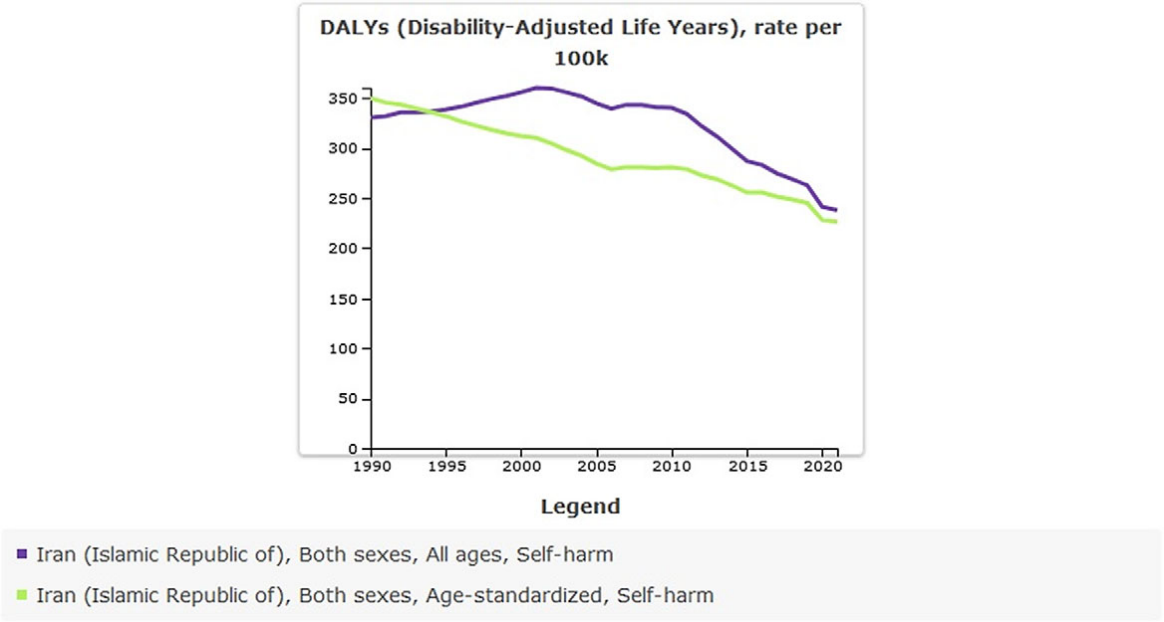
Trend in DALYs of self-harm in Iran, 1990–2021.

Age-standardized rate DALYs (per 100,000) of self-harm in 1990 was 350.18 (95% UI: 287.04–382.31) versus 226.63 (95% UI: 202.91–250.46) in 2021; the percentage change from 1990 to 2021 was −0.35% and showed a decreased DALYs. All-ages count estimates increased from 188,787 (95% UI: 148,375–206,949) in 1990 to 203,257 (95% UI: 183,865–225,136) in 2021vs. ; the percentage change from 1990 to 2021 was 0.08 (Table 3 and Figure 3).

**Table 3.** All-ages counts and age-standardized rate (per 100,000) DALYs of self-harm in Iran, stratified by provinces, 1990–2021

Location	Year								
	1990			2021			Percentage change 1990–2021		
	Value	Lower	Upper	Value	Lower	Upper	Value	Lower	Upper
Age-standardized rate DALYs (per 100,000)									
Iran	350.18	287.04	382.31	226.63	202.91	250.46	−0.35	−0.42	−0.22
Alborz	493.12	252.38	631.4	356.58	190.9	454.22	−0.28	−0.47	0.11
Ardebil	408.97	295.12	508.74	291.96	204.31	342.93	−0.29	−0.44	−0.01
Bushehr	341.13	239.41	431.86	171.32	142.1	204.44	−0.5	−0.62	−0.27
Chahar Mahaal and Bakhtiari	401.81	259.98	502.95	184.18	140.85	232.39	−0.54	−0.66	−0.33
East Azarbayejan	415.46	313.52	513.48	266.1	211.07	324.22	−0.36	−0.51	−0.14
Fars	406.88	311.49	501.78	319.17	254.46	393.1	−0.22	−0.4	0.05
Gilan	361.58	276.02	451.34	244.75	193.76	299.89	−0.32	−0.49	−0.11
Golestan	489.57	300.89	616.74	309.1	243.07	366.57	−0.37	−0.51	−0.13
Hamadan	555.83	344.06	691.64	416.68	255.41	513.88	−0.25	−0.44	−0.01
Hormozgan	356.45	275.81	454.42	260.18	207.84	320.75	−0.27	−0.47	0
Ilam	880.89	320.57	1,132.86	566.95	253.28	691.14	−0.36	−0.51	−0.08
Isfahan	178.11	139.92	268.33	144.55	114.9	198.61	−0.19	−0.4	0.1
Kerman	377.8	301.1	471.96	233.57	192.98	291.97	−0.38	−0.54	−0.16
Kermanshah	785.01	440.5	977.94	477.91	271.32	582.46	−0.39	−0.54	−0.18
Khorasan-e-Razavi	355.79	273.46	438.88	198.85	161.85	242.65	−0.44	−0.57	−0.24
Khuzestan	346.58	276.61	431.72	253.28	212.23	299.43	−0.27	−0.44	−0.04
Kohgiluyeh and Boyer-Ahmad	573.56	335.88	710.31	434.18	298.98	520.95	−0.24	−0.41	0
Kurdistan	421.82	305.53	534.14	225.02	181.42	270.28	−0.47	−0.6	−0.25
Lorestan	724.79	305.66	933.62	377.43	192.99	464.12	−0.48	−0.62	−0.25
Markazi	294.71	231.32	379.33	175.1	141.12	228.91	−0.41	−0.56	−0.19
Mazandaran	198.17	155.78	260.8	158.73	129.76	217.98	−0.2	−0.39	0.04
North Khorasan	374.07	282.56	457.11	238.5	200.23	280.85	−0.36	−0.51	−0.12
Qazvin	223.39	175.79	292.66	149.51	119.74	189.36	−0.33	−0.51	−0.1
Qom	231.49	178.3	335.87	179.69	144.44	231.96	−0.22	−0.46	0.09
Semnan	215.17	171.28	280.5	121.24	94.55	163.1	−0.44	−0.59	−0.25
Sistan and Baluchistan	190	142.26	283.89	198.58	155.68	330.4	0.05	−0.22	0.45
South Khorasan	223.24	174.56	288	158.68	133.85	196.89	−0.29	−0.45	−0.05
Tehran	170.45	130.14	232.72	106.46	83.78	153.4	−0.38	−0.53	−0.1
West Azarbayejan	539.35	371.79	675.15	334.31	234.64	396.99	−0.38	−0.52	−0.18
Yazd	200.33	160.86	283.47	120.79	95.01	167.71	−0.4	−0.56	−0.18
Zanjan	233.32	183.92	287.05	130.58	106.69	169.15	−0.44	−0.57	−0.19
All-ages counts estimates									
Iran	188,787.36	148,375.22	206,949.05	203,257.54	183,865.89	225,136.26	0.08	−0.04	0.34
Alborz	6,687.32	3,383.84	8,655.34	11,694.80	6,202.08	14,982.35	0.75	0.29	1.66
Ardebil	4,298.47	3,001.06	5,401.70	4,095.63	2,884.61	4,772.64	−0.05	−0.25	0.35
Bushehr	2,226.34	1,495.73	2,824.44	2,342.69	1,941.82	2,791.26	0.05	−0.2	0.56
Chahar Mahaal and Bakhtiari	2,572.76	1,586.08	3,295.07	1,958.68	1,497.03	2,464.93	−0.24	−0.44	0.14
East Azarbayejan	13,840.83	10,046.49	17,409.55	11,301.21	9,024.35	13,630.56	−0.18	−0.38	0.12
Fars	13,854.68	10,114.14	17,323.50	17,182.30	13,656.97	21,111.36	0.24	−0.07	0.69
Gilan	7,986.56	5,896.88	10,049.17	6,744.62	5,337.00	8,212.65	−0.16	−0.35	0.12
Golestan	6,024.01	3,505.53	7,683.93	6,186.09	4,854.80	7,301.54	0.03	−0.21	0.5
Hamadan	8,840.85	5,310.55	11,115.27	7,578.78	4,638.73	9,382.85	−0.14	−0.36	0.14
Hormozgan	2,869.23	2,165.39	3,655.39	5,477.61	4,365.06	6,742.94	0.91	0.36	1.69
Ilam	3,497.29	1,202.58	4,504.89	3,808.27	1,716.84	4,634.00	0.09	−0.18	0.64
Isfahan	6,601.83	5,114.99	9,719.58	8,094.64	6,453.58	11,114.41	0.23	−0.11	0.67
Kerman	6,300.35	4,955.29	7,911.67	8,304.06	6,854.86	10,420.84	0.32	−0.04	0.78
Kermanshah	12,517.38	6,747.37	15,817.70	10,157.17	5,823.33	12,417.83	−0.19	−0.4	0.11
Khorasan-e-Razavi	15,211.79	11,724.65	18,831.81	14,243.37	11,589.26	17,306.93	−0.06	−0.28	0.31
Khuzestan	9,906.02	7,774.19	12,392.73	12,949.45	10,892.92	15,186.51	0.31	−0.01	0.73
Kohgiluyeh and Boyer-Ahmad	2,701.46	1,511.41	3,379.37	3,666.89	2,593.28	4,395.43	0.36	0.04	0.89
Kurdistan	4,885.55	3,329.27	6,228.69	4,189.00	3,395.40	4,981.09	−0.14	−0.36	0.22
Lorestan	10,132.03	3,964.18	13,262.95	7,291.35	3,693.00	9,030.03	−0.28	−0.47	0.1
Markazi	3,375.52	2,647.18	4,259.36	2,716.78	2,183.41	3,555.95	−0.2	−0.41	0.1
Mazandaran	4,995.86	3,910.74	6,458.09	5,829.47	4,793.05	8,074.73	0.17	−0.11	0.52
North Khorasan	2,228.20	1,673.37	2,742.59	2,124.05	1,784.06	2,507.51	−0.05	−0.29	0.31
Qazvin	1,920.77	1,505.80	2,447.16	2,193.83	1,767.28	2,804.78	0.14	−0.16	0.55
Qom	1,565.97	1,188.84	2,266.52	2,768.63	2,235.96	3,585.56	0.77	0.24	1.5
Semnan	958.21	762.6	1,229.19	1,061.99	835.54	1,432.08	0.11	−0.19	0.49
Sistan and Baluchistan	2,329.67	1,674.97	3,142.36	6,234.16	4,879.94	10,396.56	1.68	0.96	2.94
South Khorasan	1,297.16	1,005.52	1,660.15	1,452.93	1,223.74	1,797.36	0.12	−0.13	0.52
Tehran	13,837.11	10,528.42	18,855.65	16,561.57	13,082.46	23,630.01	0.2	−0.11	0.73
West Azarbayejan	12,249.07	8,154.51	15,321.40	12,016.01	8,433.89	14,271.46	−0.02	−0.24	0.33
Yazd	1,230.79	976	1,682.76	1,492.94	1,178.11	2,081.85	0.21	−0.11	0.66
Zanjan	1,844.30	1,411.32	2,281.83	1,538.57	1,257.01	1,997.43	−0.17	−0.37	0.24

### Suicide mortality in Iran from 1990 to 2021

**Figure 4.**
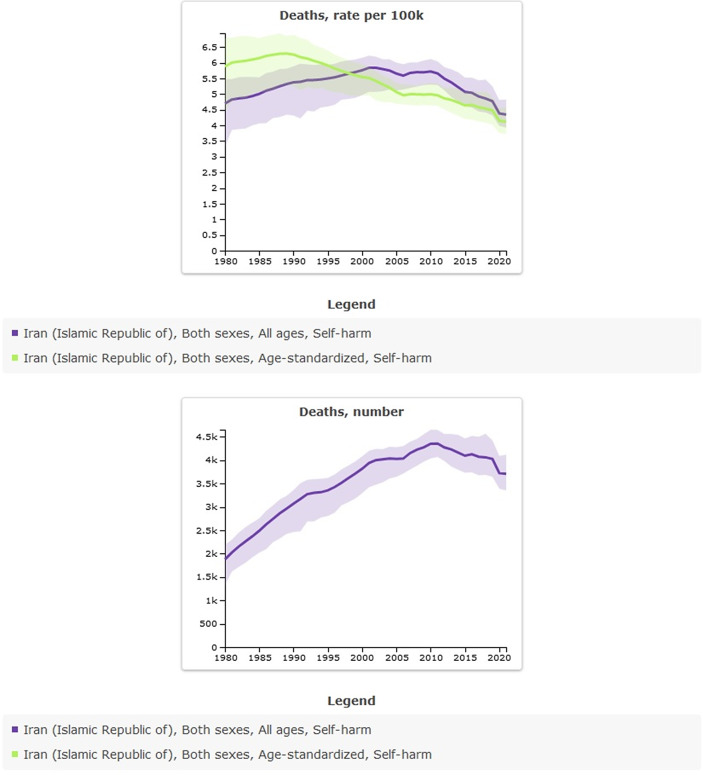
Trend in suicide mortality in Iran, 1990–2021.

Age-standardized rate (per 100,000) of suicide mortality in 1990 was 6.26 (95% UI: 5.26–6.9) versus 4.12 (95% UI: 3.72–4.58) in 2021; the percentage change from 1990 to 2021 was −0.34% and showed a decreased trend. The incidence of suicide-related mortality in 2021 amounted to 3,708 individuals. This figure indicates an increase when compared to 1990, during which the recorded number of suicide deaths was 3,069 (Table 4 and Figure 4).

**Table 4.** All-ages counts and age-standardized rate (per 100,000) of suicide mortality in Iran, stratified by provinces, 1990–2021

Location	Year								
	1990			2021			Percentage change 1990–2021		
	Value	Lower	Upper	Value	Lower	Upper	Value	Lower	Upper
Age-standardized rate suicide mortality (per 100,000)									
Iran	6.26	5.26	6.9	4.12	3.72	4.58	−0.34	−0.41	−0.19
Alborz	9.26	4.67	11.95	6.64	3.5	8.49	−0.28	−0.47	0.1
Ardebil	7.3	5.28	9.1	5.52	3.86	6.46	−0.24	−0.41	0.04
Bushehr	6.15	4.36	7.74	3.29	2.74	3.89	−0.46	−0.59	−0.23
Chahar Mahaal and Bakhtiari	7.28	4.78	9.08	3.49	2.65	4.43	−0.52	−0.64	−0.28
East Azarbayejan	7.38	5.65	9.13	4.89	3.88	5.92	−0.34	−0.49	−0.12
Fars	7.21	5.61	8.9	5.62	4.44	6.88	−0.22	−0.41	0.06
Gilan	6.71	5.22	8.29	4.56	3.58	5.56	−0.32	−0.49	−0.11
Golestan	8.59	5.36	10.68	5.67	4.44	6.69	−0.34	−0.49	−0.1
Hamadan	9.97	6.17	12.47	7.56	4.54	9.39	−0.24	−0.43	0.03
Hormozgan	6.47	4.95	8.2	4.65	3.73	5.75	−0.28	−0.48	−0.02
Ilam	16.24	5.73	20.62	10.68	4.58	12.93	−0.34	−0.49	−0.07
Isfahan	3.12	2.4	4.86	2.57	2.03	3.59	−0.18	−0.4	0.13
Kerman	6.75	5.4	8.45	4.2	3.44	5.35	−0.38	−0.54	−0.15
Kermanshah	14.09	7.97	17.46	8.64	4.81	10.57	−0.39	−0.54	−0.16
Khorasan-e-Razavi	6.4	5.06	7.95	3.67	2.98	4.46	−0.43	−0.56	−0.23
Khuzestan	6.14	4.88	7.63	4.53	3.82	5.35	−0.26	−0.43	−0.03
Kohgiluyeh and Boyer-Ahmad	9.71	5.77	12.05	7.41	5.11	8.88	−0.24	−0.4	0.01
Kurdistan	7.42	5.4	9.47	4.09	3.3	4.94	−0.45	−0.59	−0.22
Lorestan	13.2	5.59	16.99	7.28	3.57	8.93	−0.45	−0.58	−0.22
Markazi	5.23	4.14	6.98	3.14	2.51	4.19	−0.4	−0.56	−0.18
Mazandaran	3.5	2.74	4.74	2.86	2.33	3.96	−0.18	−0.37	0.08
North Khorasan	6.52	4.87	7.99	4.28	3.61	5.04	−0.34	−0.5	−0.07
Qazvin	4.06	3.22	5.58	2.83	2.27	3.63	−0.3	−0.48	−0.06
Qom	4.27	3.27	6.3	3.28	2.62	4.23	−0.23	−0.47	0.07
Semnan	3.95	3.14	5.32	2.27	1.79	3.09	−0.43	−0.58	−0.23
Sistan and Baluchistan	3.4	2.55	5.67	3.47	2.72	5.93	0.02	−0.23	0.42
South Khorasan	3.98	3.12	5.25	2.88	2.42	3.64	−0.28	−0.44	−0.02
Tehran	3.11	2.34	4.33	1.97	1.55	2.87	−0.37	−0.53	−0.07
West Azarbayejan	9.5	6.74	11.83	6.06	4.22	7.2	−0.36	−0.5	−0.15
Yazd	3.63	2.92	5.36	2.19	1.74	3.11	−0.4	−0.56	−0.17
Zanjan	4.23	3.39	5.23	2.46	2.01	3.22	−0.42	−0.55	−0.17
All-ages counts estimates									
Iran (Islamic Republic of)	3,069.66	2,463.49	3,362.63	3,708.56	3,354.15	4,117.61	0.21	0.07	0.5
Alborz	112.25	56.14	145.25	217.09	112.52	278.66	0.93	0.43	2
Ardebil	69.12	48.96	86.94	77.61	54.53	90.84	0.12	−0.11	0.58
Bushehr	35.93	24.4	45.81	42.91	35.72	50.92	0.19	−0.1	0.79
Chahar Mahaal and Bakhtiari	41.83	25.94	53.2	36.79	28.31	46.41	−0.12	−0.35	0.32
East Azarbayejan	222.88	163.9	278.89	210.69	168.22	256.38	−0.05	−0.29	0.29
Fars	223.18	166.22	277.95	305.49	243.09	375.63	0.37	0.02	0.86
Gilan	135.4	101.72	169.4	131.37	103.42	160.69	−0.03	−0.26	0.28
Golestan	96.3	56.94	121.95	112.94	89.32	134.02	0.17	−0.09	0.67
Hamadan	144.66	87.42	180.94	141.58	84.61	177.2	−0.02	−0.27	0.31
Hormozgan	47.24	35.7	61.18	94.83	75.59	117	1.01	0.45	1.8
Ilam	57.15	19.34	73.91	70.78	30.48	86.22	0.24	−0.06	0.83
Isfahan	105.42	80.84	159.8	147.13	116.7	204.98	0.4	0.01	0.93
Kerman	102.64	80.31	128.48	146.82	120.13	186.11	0.43	0.05	0.94
Kermanshah	203.33	109.99	256.36	186.67	104.82	228.98	−0.08	−0.31	0.26
Khorasan-e-Razavi	247.99	190.24	307.48	259.84	209.17	314.53	0.05	−0.21	0.44
Khuzestan	159.37	126.21	199.72	228.89	192.68	270.19	0.44	0.09	0.9
Kohgiluyeh and Boyer-Ahmad	41.58	23.27	52.13	62.25	43.42	74.27	0.5	0.15	1.08
Kurdistan	78.09	54.4	99.75	76.24	61.66	90.81	−0.02	−0.26	0.39
Lorestan	165.74	66.09	216.64	139.13	67.9	171.91	−0.16	−0.39	0.26
Markazi	54.87	42.7	70.94	49.92	39.55	66.64	−0.09	−0.33	0.26
Mazandaran	80.41	62.45	106.15	108.66	88.26	152.86	0.35	0.03	0.79
North Khorasan	35.51	26.21	43.78	37.92	31.95	44.84	0.07	−0.21	0.49
Qazvin	31.46	24.44	40.78	41.29	33.04	53.25	0.31	−0.02	0.8
Qom	25.75	19.64	37.82	49.67	39.95	64.19	0.93	0.34	1.75
Semnan	16.21	12.86	21.11	19.59	15.53	26.77	0.21	−0.12	0.63
Sistan and Baluchistan	37.63	27.62	54.31	103.54	80.93	176.23	1.75	1.02	2.96
South Khorasan	21.33	16.44	27.63	26.09	21.8	32.83	0.22	−0.07	0.65
Tehran	231.21	173.85	319.4	312.09	243.11	451.58	0.35	−0.01	1
West Azarbayejan	194.55	131.62	243.98	214.92	149.49	257.61	0.1	−0.14	0.49
Yazd	20.4	16.21	28.9	26.7	21.14	37.83	0.31	−0.05	0.81
Zanjan	30.27	23.66	37.37	29.13	23.64	38.12	−0.04	−0.27	0.39

### The sex-specific burden of self-harm and suicide mortality in Iran

**Figure 5.**
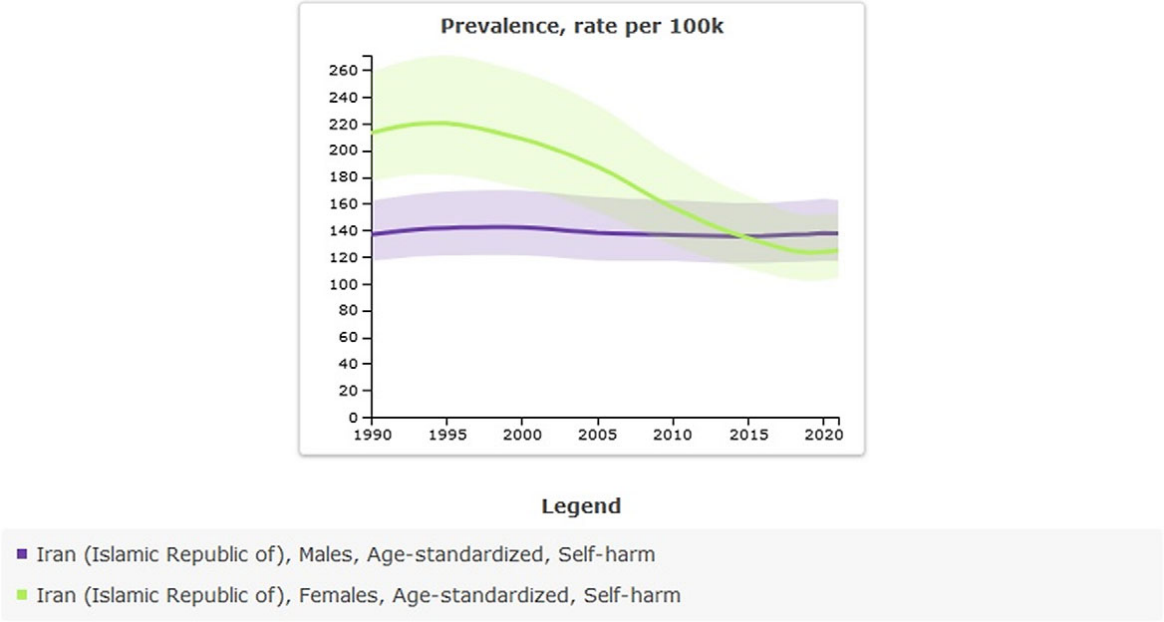
Sex-specific prevalence of self-harm in Iran, 1990–2021.

There are differences between males and females in the prevalence, incidence, DALYs and suicide mortality of self-harm. In 1990, the prevalence of self-harm was lower in males than in females. For males, the age-standardized rate (per 100,000) was 136.66 (95% UI: 116.78–162.01); for females, it was 212.79 (95% UI: 176.25–258.02). In 2021, the prevalence of self-harm was higher in males (137.62 per 100,000) than in females (124.82 per 100,000). Since 2015, a notable shift in the prevalence rates of self-harm among men and women has been observed, signaling an epidemiological transformation. By 2021, the incidence of self-harm reached a total of 64,913 cases among males (95% UI: 54,867–76,963) and 57,867 cases among females (95% UI: 48,020–70,468) (Table 5 and Figure 5).

**Table 5.** Sex-specific prevalence, incidence, and DALYs of self-harm in Iran, stratified by provinces, 1990–2021

Location	Sex	Year								
		1990			2021			Percentage change 1990–2021		
		Value	Lower	Upper	Value	Lower	Upper	Value	Lower	Upper
Age-standardized rate prevalence (per 100,000)										
Iran (Islamic Republic of)	Males	136.66	116.78	162.01	137.62	116.74	162.53	0.01	−0.03	0.04
Alborz	Males	163.83	138.08	193.6	170.83	143.83	203.19	0.04	0.01	0.08
Ardebil	Males	147.11	125.39	174.27	146.43	123.87	173.2	0	−0.05	0.04
Bushehr	Males	108.32	93.34	127.56	115.08	99.08	134.96	0.06	0.03	0.1
Chahar Mahaal and Bakhtiari	Males	141.49	121.28	167.54	122.41	104.08	144.75	−0.13	−0.17	−0.11
East Azarbayejan	Males	146.14	122.95	175.12	150.01	126.79	177.18	0.03	−0.04	0.09
Fars	Males	156.62	132.69	186.4	154.82	130.19	182.87	−0.01	−0.06	0.03
Gilan	Males	129.48	109.09	154.08	142.85	121.01	168.34	0.1	0.05	0.16
Golestan	Males	132.94	112.99	157.58	138.12	116.27	163.85	0.04	0	0.08
Hamadan	Males	183.67	154.66	217.44	178.33	150.46	210.48	−0.03	−0.09	0.02
Hormozgan	Males	136.49	116.77	160.3	143.66	120.47	170.41	0.05	0.02	0.09
Ilam	Males	174.94	147.95	207.73	185.15	155.1	219.47	0.06	0.01	0.11
Isfahan	Males	120.93	104.21	142.67	131.21	110.32	156.01	0.08	0.05	0.13
Kerman	Males	130.06	110.03	155.79	135.89	114.57	161.69	0.04	0	0.09
Kermanshah	Males	213.47	180.1	252.98	171.91	144.46	202.43	−0.19	−0.24	−0.16
Khorasan-e-Razavi	Males	120.01	101.73	142.93	131.06	111.92	154.32	0.09	0.04	0.15
Khuzestan	Males	125.51	107.76	147.84	135.26	114.71	160.87	0.08	0.04	0.12
Kohgiluyeh and Boyer-Ahmad	Males	152.92	128.2	185.68	161.33	133.97	191.2	0.05	0	0.11
Kurdistan	Males	144.67	124.12	171.59	133.17	112.12	157.88	−0.08	−0.12	−0.04
Lorestan	Males	170.14	144.18	200.88	162.63	136.65	191.88	−0.04	−0.08	−0.01
Markazi	Males	153.06	129.43	183.8	131.17	110.5	154.49	−0.14	−0.19	−0.1
Mazandaran	Males	106.78	91.57	125.97	129.96	110.33	154.04	0.22	0.17	0.26
North Khorasan	Males	123.89	106.34	147.15	125.66	107.34	149.21	0.01	−0.03	0.06
Qazvin	Males	94.46	81.32	111.76	127.18	107.98	149.52	0.35	0.3	0.41
Qom	Males	129.43	110.25	153.63	130.34	109.48	155.05	0.01	−0.04	0.06
Semnan	Males	108.7	93.72	127.54	123.29	104.92	144.83	0.13	0.1	0.17
Sistan and Baluchistan	Males	98.52	85.6	115.04	127.44	108.69	150.3	0.29	0.25	0.35
South Khorasan	Males	99.77	85.85	116.51	124.17	106.32	146.05	0.24	0.19	0.3
Tehran	Males	140.72	121.56	164.86	120.41	103.19	141.56	−0.14	−0.17	−0.12
West Azarbayejan	Males	136.83	114.97	165.12	152.28	128.35	178.97	0.11	0.05	0.18
Yazd	Males	106.22	91.71	125.49	118.59	100.71	140.09	0.12	0.08	0.17
Zanjan	Males	94.22	81.02	111.67	112.97	96.35	132.83	0.2	0.16	0.25
Iran (Islamic Republic of)	Females	212.79	176.25	258.02	124.82	104.03	151.66	−0.41	−0.44	−0.39
Alborz	Females	194.62	160.52	237.67	133.38	109.69	162.29	−0.31	−0.34	−0.29
Ardebil	Females	262.38	218.03	320.49	132.92	111.12	159.89	−0.49	−0.53	−0.46
Bushehr	Females	263.56	218.14	319.91	137.95	115.98	164.82	−0.48	−0.51	−0.44
Chahar Mahaal and Bakhtiari	Females	287.49	237.05	346.59	142.37	117.99	172.09	−0.5	−0.54	−0.48
East Azarbayejan	Females	206.17	168.84	255.19	125.54	104.22	153.25	−0.39	−0.42	−0.36
Fars	Females	268.48	220.89	329.47	146.49	121.12	178.87	−0.45	−0.48	−0.43
Gilan	Females	187.13	153.98	227.69	125.22	103.71	152.34	−0.33	−0.36	−0.3
Golestan	Females	325.86	267.47	409.23	157.58	131.42	190.88	−0.52	−0.55	−0.48
Hamadan	Females	235.8	193.77	293.79	142.45	117.46	173.31	−0.4	−0.43	−0.37
Hormozgan	Females	196.78	163.41	237.7	108.38	91.43	131.41	−0.45	−0.48	−0.42
Ilam	Females	391.63	322.86	477.01	191.76	159.36	229.14	−0.51	−0.54	−0.48
Isfahan	Females	147.17	122.35	179.41	102.96	86.62	124.11	−0.3	−0.34	−0.27
Kerman	Females	201.38	166.13	245.48	126.81	105.55	155.48	−0.37	−0.4	−0.34
Kermanshah	Females	396.03	327.12	494.24	186.02	152.78	228.87	−0.53	−0.56	−0.5
Khorasan-e-Razavi	Females	219.32	181.16	269.09	119.19	99.32	143.53	−0.46	−0.49	−0.42
Khuzestan	Females	247.81	204.57	301.81	145.62	119.92	177.73	−0.41	−0.44	−0.38
Kohgiluyeh and Boyer-Ahmad	Females	307.75	252.04	385.9	177.37	145.18	217.53	−0.42	−0.45	−0.39
Kurdistan	Females	295.53	243.08	367.49	129.3	107.95	156.02	−0.56	−0.59	−0.53
Lorestan	Females	339.58	281.1	417.86	185.75	153.94	224.89	−0.45	−0.48	−0.42
Markazi	Females	172.34	143.2	209.3	97.89	82.65	117.82	−0.43	−0.47	−0.4
Mazandaran	Females	161.91	133.47	198.03	114.71	94.21	139.21	−0.29	−0.32	−0.26
North Khorasan	Females	288.87	239.4	358.38	154.63	128.55	187.04	−0.46	−0.49	−0.44
Qazvin	Females	151.48	126.66	182.54	105.33	88.65	127.22	−0.3	−0.33	−0.28
Qom	Females	117.62	99.64	140.8	85.98	73.07	103.62	−0.27	−0.3	−0.24
Semnan	Females	137.72	114.97	167.14	95.06	80.27	114.2	−0.31	−0.34	−0.28
Sistan and Baluchistan	Females	134.15	112.85	160.44	115.94	97.34	139.55	−0.14	−0.16	−0.11
South Khorasan	Females	178.25	148.6	216.38	120.68	101.08	147.06	−0.32	−0.35	−0.3
Tehran	Females	153.7	129.42	183.73	98.65	83.33	118.84	−0.36	−0.39	−0.33
West Azarbayejan	Females	285.56	234.25	358.33	159.06	131.37	194.74	−0.44	−0.47	−0.42
Yazd	Females	174.02	145.29	210.98	105.78	88.33	127.36	−0.39	−0.43	−0.37
Zanjan	Females	155.04	127.61	189.14	101.03	84.81	121.27	−0.35	−0.39	−0.32
All-ages prevalence counts estimates										
Iran (Islamic Republic of)	Males	25,980.61	21,921.03	31,313.86	64,913.99	54,867.53	76,963.05	1.5	1.36	1.63
Alborz	Males	799.12	666.04	960.89	3,004.47	2,512.81	3,580.00	2.76	2.54	2.98
Ardebil	Males	526.13	442.47	628.02	1,066.37	896.7	1,266.74	1.03	0.9	1.15
Bushehr	Males	232.98	197.84	280.1	746.01	634.99	883.77	2.2	2.03	2.37
Chahar Mahaal and Bakhtiari	Males	308.66	261.85	369.18	658.52	554.24	787.27	1.13	1.01	1.24
East Azarbayejan	Males	1,727.24	1,439.06	2,099.40	3,566.44	3,006.81	4,224.22	1.06	0.89	1.23
Fars	Males	1,842.56	1,541.73	2,227.87	4,527.43	3,782.49	5,373.18	1.46	1.28	1.62
Gilan	Males	1,064.00	889.05	1,286.54	2,381.49	2,017.29	2,802.77	1.24	1.08	1.4
Golestan	Males	543.91	459.05	656.48	1,396.89	1,169.92	1,669.31	1.57	1.45	1.71
Hamadan	Males	1,037.36	870.46	1,245.50	1,819.30	1,531.40	2,154.08	0.75	0.61	0.89
Hormozgan	Males	395.57	333.8	470.5	1,367.40	1,133.36	1,646.31	2.46	2.29	2.63
Ilam	Males	233.76	196.32	281.15	623.48	518.14	745.61	1.67	1.48	1.86
Isfahan	Males	1,583.45	1,347.54	1,896.86	4,184.50	3,512.20	4,998.22	1.64	1.51	1.79
Kerman	Males	771.08	646.96	936.16	2,417.17	2,016.64	2,890.05	2.13	1.95	2.33
Kermanshah	Males	1,188.21	993.95	1,432.18	1,974.55	1,651.23	2,333.74	0.66	0.54	0.78
Khorasan-e-Razavi	Males	1,833.49	1,550.81	2,204.80	4,647.60	3,941.63	5,499.90	1.53	1.37	1.7
Khuzestan	Males	1,194.35	1,014.86	1,439.53	3,419.90	2,874.35	4,087.69	1.86	1.72	2.02
Kohgiluyeh and Boyer-Ahmad	Males	219.1	182.4	270.9	646.6	532.49	777.9	1.95	1.74	2.16
Kurdistan	Males	578.62	491.99	692.14	1,280.78	1,072.53	1,526.19	1.21	1.09	1.34
Lorestan	Males	811.01	682.51	967.64	1,543.82	1,288.03	1,833.60	0.9	0.8	1.01
Markazi	Males	617.56	518.61	748.5	1,128.17	948.56	1,336.84	0.83	0.7	0.96
Mazandaran	Males	919.91	780.92	1,100.78	2,800.21	2,371.82	3,320.58	2.04	1.88	2.23
North Khorasan	Males	255.36	216.95	307.29	562.07	477.11	670.29	1.2	1.06	1.35
Qazvin	Males	283.28	241.67	340.29	970.2	817.79	1,146.22	2.42	2.23	2.65
Qom	Males	309.33	260.62	375.88	992.11	825.86	1,188.43	2.21	2	2.46
Semnan	Males	186.59	160	221.95	537.86	454.96	635.85	1.88	1.75	2.03
Sistan and Baluchistan	Males	419.79	362.38	494.65	1,494.14	1,261.12	1,780.24	2.56	2.42	2.73
South Khorasan	Males	223.13	192.09	261.35	542.42	460.96	643.26	1.43	1.31	1.56
Tehran	Males	4,320.45	3,699.32	5,140.16	10,377.91	8,885.78	12,242.71	1.4	1.28	1.51
West Azarbayejan	Males	1,044.44	873.45	1,276.68	2,796.59	2,335.12	3,312.87	1.68	1.48	1.9
Yazd	Males	245.46	210.04	292.23	751.43	634.95	893.43	2.06	1.92	2.24
Zanjan	Males	264.71	226.32	316.18	688.16	584.17	815.08	1.6	1.48	1.76
Iran (Islamic Republic of)	Females	39,854.26	32,912.27	49,170.48	57,867.77	48,020.96	70,468.16	0.45	0.36	0.53
Alborz	Females	897.71	735.44	1,117.21	2,255.41	1,843.73	2,758.72	1.51	1.38	1.64
Ardebil	Females	910.45	751.28	1,133.08	953.21	794.49	1,149.59	0.05	−0.03	0.12
Bushehr	Females	571.59	468.55	710.8	827.09	689.69	993.68	0.45	0.32	0.56
Chahar Mahaal and Bakhtiari	Females	605.73	496.13	746.28	755.37	622.38	918.81	0.25	0.15	0.33
East Azarbayejan	Females	2,376.11	1,938.71	2,969.95	2,899.41	2,405.31	3,533.16	0.22	0.13	0.3
Fars	Females	3,092.95	2,512.98	3,879.40	4,191.23	3,453.43	5,132.54	0.36	0.27	0.44
Gilan	Females	1,579.18	1,291.76	1,943.30	2,058.57	1,709.06	2,493.13	0.3	0.23	0.38
Golestan	Females	1,355.35	1,099.45	1,738.65	1,632.84	1,356.21	1,986.89	0.2	0.09	0.32
Hamadan	Females	1,304.44	1,062.72	1,644.82	1,413.44	1,166.56	1,719.03	0.08	0.01	0.16
Hormozgan	Females	539.4	446.17	662.17	995.01	829.73	1,212.26	0.84	0.73	0.96
Ilam	Females	480.97	388.86	602.86	644.05	532.2	776.04	0.34	0.23	0.45
Isfahan	Females	1,864.13	1,541.57	2,294.25	3,176.54	2,668.91	3,838.50	0.7	0.58	0.8
Kerman	Females	1,172.83	966.64	1,449.53	2,144.63	1,771.82	2,645.44	0.83	0.73	0.91
Kermanshah	Females	2,097.00	1,707.56	2,682.03	2,154.17	1,763.87	2,652.71	0.03	−0.05	0.11
Khorasan-e-Razavi	Females	3,331.09	2,728.24	4,131.31	4,232.83	3,510.21	5,115.22	0.27	0.17	0.36
Khuzestan	Females	2,340.69	1,917.52	2,916.10	3,636.86	2,971.03	4,474.76	0.55	0.47	0.63
Kohgiluyeh and Boyer-Ahmad	Females	416.87	337.4	533.86	687.17	556.58	853.49	0.65	0.54	0.76
Kurdistan	Females	1,148.07	937.16	1,447.30	1,215.41	1,010.46	1,471.07	0.06	−0.04	0.14
Lorestan	Females	1,554.36	1,273.86	1,954.09	1,804.10	1,488.74	2,188.69	0.16	0.08	0.25
Markazi	Females	709.95	589.45	871.35	822.79	694.96	990.55	0.16	0.06	0.23
Mazandaran	Females	1,448.63	1,184.90	1,810.57	2,441.21	2,001.92	2,965.36	0.69	0.58	0.79
North Khorasan	Females	599.95	494.62	756.91	694.63	575.09	843.12	0.16	0.08	0.23
Qazvin	Females	452.71	377.39	554.24	778.39	652.81	944.25	0.72	0.62	0.81
Qom	Females	268.75	226.52	323.73	626.73	529.19	758.85	1.33	1.2	1.46
Semnan	Females	236.57	197.03	289.77	407.88	342	493.68	0.72	0.62	0.81
Sistan and Baluchistan	Females	547.85	460.26	667.46	1,394.56	1,160.24	1,705.03	1.55	1.45	1.63
South Khorasan	Females	391.6	327.67	478.47	533.79	445.96	652.73	0.36	0.29	0.42
Tehran	Females	4,583.38	3,826.56	5,549.04	8,358.90	7,024.47	10,099.26	0.82	0.71	0.91
West Azarbayejan	Females	2,149.33	1,741.77	2,737.79	2,890.52	2,378.10	3,553.10	0.34	0.25	0.44
Yazd	Females	390.49	324.71	477.85	630.17	523.85	762.77	0.61	0.51	0.7
Zanjan	Females	436.14	357.65	541.26	610.84	510.35	735.01	0.4	0.29	0.49
Age-standardized rate incidence (per 100,000)										
Iran (Islamic Republic of)	Males	39.72	32.38	48.02	42.9	33.59	53.18	0.08	0	0.15
Alborz	Males	48.1	38.96	59.03	54.42	42.69	68.08	0.13	0.03	0.23
Ardebil	Males	41.99	34.41	51.28	45.82	35.69	57.3	0.09	−0.02	0.19
Bushehr	Males	31.21	25.45	38.25	36.19	28.39	44.41	0.16	0.05	0.28
Chahar Mahaal and Bakhtiari	Males	41.53	33.68	50.62	39.02	30.82	48.73	−0.06	−0.15	0.03
East Azarbayejan	Males	41.09	33.28	50.27	46.58	36.29	58.7	0.13	0.02	0.27
Fars	Males	45.45	37	55.34	48.4	37.83	60.3	0.06	−0.03	0.16
Gilan	Males	40.3	32.91	48.8	45.19	35.38	56.99	0.12	0	0.23
Golestan	Males	37.94	30.66	46.1	42.16	32.92	53.06	0.11	0.02	0.23
Hamadan	Males	52.45	42.43	64.45	55.56	43.28	68.82	0.06	−0.04	0.16
Hormozgan	Males	38.5	31.11	46.72	43.79	34.36	54.93	0.14	0.03	0.24
Ilam	Males	50.27	41.26	61.28	58.1	45	72.6	0.16	0.04	0.27
Isfahan	Males	35.49	28.47	43.13	41.06	32.74	50.77	0.16	0.06	0.26
Kerman	Males	36.98	29.85	44.73	41.55	32.1	52.04	0.12	0.01	0.24
Kermanshah	Males	61.34	50.34	74.75	53.38	41.59	66.15	−0.13	−0.21	−0.04
Khorasan-e-Razavi	Males	33.79	27.52	40.97	40.27	31.53	50.11	0.19	0.08	0.31
Khuzestan	Males	36.08	28.98	44.22	41.66	32.7	51.78	0.15	0.07	0.24
Kohgiluyeh and Boyer-Ahmad	Males	43.15	34.7	53.23	50.59	38.96	64.31	0.17	0.05	0.31
Kurdistan	Males	41.32	33.83	50.2	40.97	31.75	51.37	−0.01	−0.11	0.09
Lorestan	Males	48.83	39.9	59.32	51.73	40.34	63.26	0.06	−0.03	0.15
Markazi	Males	44	35.67	52.97	40.57	31.82	51.58	−0.08	−0.16	0.01
Mazandaran	Males	31.42	25.57	38.43	41.07	32.35	51.67	0.31	0.21	0.43
North Khorasan	Males	34.68	28.02	42.38	38.13	30	47.22	0.1	−0.01	0.2
Qazvin	Males	26.83	21.79	32.55	39.76	31.53	49.6	0.48	0.35	0.64
Qom	Males	36.96	30.05	44.49	40.46	31.41	50.45	0.09	0	0.2
Semnan	Males	31.35	25.23	38.06	38.6	29.97	48.37	0.23	0.13	0.33
Sistan and Baluchistan	Males	27.74	21.92	34.25	37.47	28.93	47.15	0.35	0.25	0.46
South Khorasan	Males	28.03	22.76	33.88	38.28	30.21	47.44	0.37	0.24	0.49
Tehran	Males	41.75	33.7	50.54	38.31	29.94	47.4	−0.08	−0.17	0
West Azarbayejan	Males	38.43	31.11	47.37	47.1	36.98	58.71	0.23	0.12	0.36
Yazd	Males	30.86	25.05	37.32	36.93	28.81	46.11	0.2	0.08	0.32
Zanjan	Males	28.29	22.73	34.5	35.28	28.15	43.95	0.25	0.12	0.35
Iran (Islamic Republic of)	Females	75.31	60.81	92.21	49.28	38.52	62	−0.35	−0.39	−0.31
Alborz	Females	69.93	56.65	86.89	53.88	41.9	68.12	−0.23	−0.31	−0.16
Ardebil	Females	90.98	73.02	112.32	52.35	40.71	65.42	−0.42	−0.48	−0.37
Bushehr	Females	93.4	75.67	114.33	55.48	43.55	69.35	−0.41	−0.46	−0.35
Chahar Mahaal and Bakhtiari	Females	102.83	83.08	126.35	58.52	46.08	73.73	−0.43	−0.48	−0.37
East Azarbayejan	Females	70.01	55.34	86.87	48.49	37.42	62.21	−0.31	−0.38	−0.24
Fars	Females	94.77	76.48	116.55	57.71	44.92	73.28	−0.39	−0.44	−0.33
Gilan	Females	71.35	57.66	87.03	50.4	39.7	63.99	−0.29	−0.36	−0.23
Golestan	Females	112.34	90.13	138.03	60.77	47.93	75.37	−0.46	−0.51	−0.41
Hamadan	Females	80.68	65.52	99.61	55.05	42.47	69.87	−0.32	−0.39	−0.25
Hormozgan	Females	66.64	53.85	82.32	41.61	32.16	52.86	−0.38	−0.43	−0.3
Ilam	Females	139.23	113.16	168.87	77.78	61.73	95.92	−0.44	−0.49	−0.39
Isfahan	Females	52.12	41.67	65.11	40.98	31.81	51.61	−0.21	−0.29	−0.15
Kerman	Females	68.94	55.26	85.3	48.36	37.41	61.5	−0.3	−0.37	−0.22
Kermanshah	Females	138.04	112.06	169.9	72.75	57.16	90.73	−0.47	−0.52	−0.42
Khorasan-e-Razavi	Females	74.15	59.57	92.46	45.74	35.58	57.57	−0.38	−0.44	−0.33
Khuzestan	Females	87.24	69.38	108.88	56.74	43.87	71.17	−0.35	−0.41	−0.29
Kohgiluyeh and Boyer-Ahmad	Females	107.69	87.03	132.6	71.09	55.83	89.33	−0.34	−0.4	−0.28
Kurdistan	Females	101.26	81.7	122.94	50.66	39.87	64.11	−0.5	−0.55	−0.45
Lorestan	Females	119.17	96.36	147.34	75.39	59.05	93.69	−0.37	−0.42	−0.32
Markazi	Females	59.36	47.63	73.22	38.29	30.12	48.37	−0.35	−0.41	−0.29
Mazandaran	Females	58	46.51	71.38	46.12	35.57	57.86	−0.2	−0.28	−0.13
North Khorasan	Females	97.64	78.89	119.7	59.16	46.52	74.68	−0.39	−0.45	−0.34
Qazvin	Females	52.13	41.71	64.24	41.25	32.29	52.19	−0.21	−0.29	−0.13
Qom	Females	40.48	32.78	50.57	33.92	25.83	43.45	−0.16	−0.26	−0.08
Semnan	Females	47.48	38.05	59.29	37.41	28.89	47.26	−0.21	−0.3	−0.14
Sistan and Baluchistan	Females	44.55	35.73	54.64	41.62	32	53.52	−0.07	−0.15	0.03
South Khorasan	Females	60.06	48.63	74.43	46.46	36.04	58.83	−0.23	−0.3	−0.16
Tehran	Females	56.12	45.41	69.69	40.44	31.08	51.82	−0.28	−0.35	−0.21
West Azarbayejan	Females	97.25	78.63	119.79	61.7	48.35	76.94	−0.37	−0.43	−0.3
Yazd	Females	60.92	48.29	74.42	41.81	32.46	53.5	−0.31	−0.37	−0.25
Zanjan	Females	56.57	45.39	69.97	39.65	30.62	50.19	−0.3	−0.37	−0.23
All-ages incidence counts estimates										
Iran (Islamic Republic of)	Males	10,863.93	8,683.10	13,557.19	19,891.51	15,639.20	24,678.36	0.83	0.61	1.09
Alborz	Males	349.13	277.04	435.04	919.83	722.14	1,136.48	1.63	1.3	2.04
Ardebil	Males	227.44	178.89	286.2	335.04	260.71	417.72	0.47	0.24	0.75
Bushehr	Males	103.53	81.91	129.82	265.07	206.41	327.44	1.56	1.22	1.96
Chahar Mahaal and Bakhtiari	Males	135.96	107.26	171.83	212.83	168.2	266.23	0.57	0.35	0.82
East Azarbayejan	Males	696.7	552.65	887.62	1,035.48	810.01	1,287.90	0.49	0.26	0.77
Fars	Males	787.85	623.31	989.99	1,385.60	1,080.34	1,737.76	0.76	0.52	1.04
Gilan	Males	451.4	360.52	562.08	637.71	504	794.34	0.41	0.19	0.66
Golestan	Males	229.91	179.83	287.42	431.8	338.18	538.01	0.88	0.64	1.13
Hamadan	Males	439.32	344.46	555.95	523.41	408.46	649.82	0.19	0.02	0.39
Hormozgan	Males	160.93	126.8	200.21	480.39	370.69	606.76	1.99	1.59	2.38
Ilam	Males	105.95	83.72	133.72	200.72	155.85	251.21	0.89	0.6	1.23
Isfahan	Males	671.02	528.33	836.46	1,181.98	935.71	1,457.97	0.76	0.51	1.05
Kerman	Males	317.11	246.88	391.76	785.62	604.24	981.57	1.48	1.13	1.88
Kermanshah	Males	503.87	403.65	633.65	587.22	460.29	726.85	0.17	0	0.38
Khorasan-e-Razavi	Males	730.31	582.84	914.52	1,480.67	1,164.70	1,829.01	1.03	0.74	1.36
Khuzestan	Males	527.11	411.93	672.32	1,109.54	861.75	1,382.74	1.1	0.85	1.41
Kohgiluyeh and Boyer-Ahmad	Males	100.74	78.91	129.96	223.81	171.48	285.39	1.22	0.86	1.62
Kurdistan	Males	239.46	190.86	298.92	395.72	304.63	494.97	0.65	0.4	0.95
Lorestan	Males	355.86	281.4	447.12	506.42	395.45	624.25	0.42	0.25	0.65
Markazi	Males	253.53	200.4	317.66	321.6	252.86	400.58	0.27	0.09	0.47
Mazandaran	Males	390.13	308.76	487.34	780.42	615.57	967.57	1	0.72	1.35
North Khorasan	Males	102.75	81.65	129.89	175.36	136.51	218.26	0.71	0.47	0.98
Qazvin	Males	119.73	94.42	151.06	301.01	237.57	374.02	1.51	1.14	2
Qom	Males	132.81	105.17	163.95	316.62	245.77	394.79	1.38	1.06	1.73
Semnan	Males	73.01	57.85	90.05	173.62	133.57	217.61	1.38	1.1	1.7
Sistan and Baluchistan	Males	169.5	130.86	215.52	595.14	452.38	764.92	2.51	2.15	2.88
South Khorasan	Males	81.25	64.94	100.04	181.89	142.49	226.33	1.24	0.98	1.55
Tehran	Males	1,752.87	1,391.40	2,166.57	3,005.67	2,368.74	3,694.62	0.71	0.51	0.95
West Azarbayejan	Males	441.43	347.17	562.15	889.18	698.07	1,109.11	1.01	0.74	1.35
Yazd	Males	99.38	78.97	125	239.72	185.31	297.94	1.41	1.12	1.75
Zanjan	Males	113.93	89.73	142.92	212.42	168.78	265.49	0.86	0.54	1.18
Iran (Islamic Republic of)	Females	21,219.21	16,839.31	26,855.28	21,017.07	16,503.98	26,128.95	−0.01	−0.11	0.12
Alborz	Females	503.71	396.1	628.71	818.8	639.42	1,013.54	0.63	0.43	0.87
Ardebil	Females	498.76	389.38	629.6	339.64	268.61	422.02	−0.32	−0.41	−0.21
Bushehr	Females	325.46	258.8	412.46	353.89	276.8	443.47	0.09	−0.04	0.24
Chahar Mahaal and Bakhtiari	Females	348.91	273	436.97	294.38	232	367.34	−0.16	−0.27	−0.03
East Azarbayejan	Females	1,208.35	934.52	1,536.14	954.99	740.22	1,186.94	−0.21	−0.31	−0.1
Fars	Females	1,680.46	1,330.32	2,108.27	1,481.23	1,167.81	1,854.87	−0.12	−0.22	0.01
Gilan	Females	838.7	668.31	1,040.75	643.01	510.03	785.47	−0.23	−0.33	−0.12
Golestan	Females	732.93	577.22	929.77	594.32	468.88	729.33	−0.19	−0.29	−0.07
Hamadan	Females	669.53	528.38	850.1	465.04	363.84	578.97	−0.31	−0.4	−0.19
Hormozgan	Females	288.03	227.28	365.29	421.36	327.17	525.13	0.46	0.29	0.67
Ilam	Females	293.75	234.37	372.99	249.02	197.19	307.94	−0.15	−0.28	0
Isfahan	Females	974.47	765.58	1,248.17	1,090.00	852.83	1,341.85	0.12	−0.03	0.3
Kerman	Females	611.74	482.85	780.36	809.32	632.07	1,023.44	0.32	0.16	0.53
Kermanshah	Females	1,130.91	888.73	1,409.42	728.65	576.29	904.08	−0.36	−0.44	−0.26
Khorasan-e-Razavi	Females	1,698.79	1,340.32	2,177.33	1,576.79	1,231.59	1,974.26	−0.07	−0.19	0.04
Khuzestan	Females	1,316.03	1,032.12	1,684.08	1,408.26	1,089.00	1,753.13	0.07	−0.06	0.21
Kohgiluyeh and Boyer-Ahmad	Females	256.64	201.84	328.19	282.52	219.99	354.32	0.1	−0.05	0.28
Kurdistan	Females	614.05	482.59	767.4	446.05	354.05	556.64	−0.27	−0.38	−0.16
Lorestan	Females	892.95	704.49	1,138.94	682.78	535.33	843.42	−0.24	−0.34	−0.12
Markazi	Females	358.74	281.78	458.42	279.89	221.22	348.1	−0.22	−0.33	−0.09
Mazandaran	Females	779.54	616.65	980.67	796.95	622.31	975.06	0.02	−0.11	0.19
North Khorasan	Females	303.61	238.02	379.34	254.41	201.06	316.96	−0.16	−0.26	−0.05
Qazvin	Females	241.75	189.38	305.89	279.86	219.89	347.65	0.16	0	0.36
Qom	Females	144.86	112.99	185.03	249.94	191.72	314.54	0.73	0.49	0.98
Semnan	Females	110.84	87.3	141.23	154.39	118.55	194.87	0.39	0.2	0.59
Sistan and Baluchistan	Females	298.76	233.08	384.36	653.6	495.96	847.86	1.19	0.97	1.45
South Khorasan	Females	187.09	148.51	238.84	201.48	157.76	253.72	0.08	−0.03	0.2
Tehran	Females	2,338.70	1,866.34	2,988.74	2,981.26	2,316.35	3,738.27	0.27	0.12	0.47
West Azarbayejan	Females	1,138.65	893.93	1,434.19	1,059.09	840.84	1,303.36	−0.07	−0.19	0.06
Yazd	Females	191.87	149.79	241.73	249.24	193.39	314.46	0.3	0.16	0.45
Zanjan	Females	240.65	189.64	307.1	216.89	168.89	269.18	−0.1	−0.22	0.05
Age-standardized rate DALYs (per 100,000)										
Iran (Islamic Republic of)	Males	394.97	339.53	445.59	325.29	277.26	357.39	−0.18	−0.28	−0.03
Alborz	Males	694.07	289.42	940.95	568.33	270.5	740.65	−0.18	−0.44	0.29
Ardebil	Males	437.62	324.74	559.98	427.56	283.87	514.44	−0.02	−0.31	0.35
Bushehr	Males	255.66	188.19	363.54	185.59	145.2	236.09	−0.27	−0.48	0.02
Chahar Mahaal and Bakhtiari	Males	378.01	278.62	484.44	233.23	183.4	295.33	−0.38	−0.56	−0.08
East Azarbayejan	Males	529.26	367.79	700.6	400.04	291.82	502.15	−0.24	−0.45	0.07
Fars	Males	427.21	321.88	568.62	452.77	340.94	578.21	0.06	−0.27	0.5
Gilan	Males	446.55	325.39	591.94	374.08	278.67	478.36	−0.16	−0.41	0.18
Golestan	Males	374.4	278.02	490.89	389.87	310.2	486.92	0.04	−0.24	0.42
Hamadan	Males	740.03	449.09	976.54	667.57	366.81	849.16	−0.1	−0.37	0.23
Hormozgan	Males	443.71	318.94	591.52	426.21	321.3	542.79	−0.04	−0.33	0.42
Ilam	Males	803.7	358.86	1,068.51	783.79	363.66	981.94	−0.02	−0.3	0.39
Isfahan	Males	226.08	166.18	348.91	227.97	177.84	302.1	0.01	−0.31	0.5
Kerman	Males	431.89	331.87	578.97	321.77	255.42	417.4	−0.25	−0.47	0.09
Kermanshah	Males	831.54	543.57	1,084.82	643.3	361.75	802.83	−0.23	−0.45	0.07
Khorasan-e-Razavi	Males	380.64	283.59	508.14	280.69	222.07	351.07	−0.26	−0.49	0.05
Khuzestan	Males	327.9	248.49	444.45	332.82	265	409.69	0.01	−0.26	0.43
Kohgiluyeh and Boyer-Ahmad	Males	550.17	389.45	714.78	538.36	386.37	669.28	−0.02	−0.31	0.42
Kurdistan	Males	396.96	283.42	525.24	313.95	244.99	393.81	−0.21	−0.45	0.19
Lorestan	Males	706.38	373.4	925.06	506.66	268.65	637.51	−0.28	−0.48	0.02
Markazi	Males	420.77	313.47	544.22	286.03	224.74	361.11	−0.32	−0.53	−0.05
Mazandaran	Males	213.73	158.55	329.37	225.5	178.65	310.47	0.06	−0.26	0.54
North Khorasan	Males	310.01	232.57	425.74	279.62	224.36	350.7	−0.1	−0.35	0.3
Qazvin	Males	238.23	180.7	360.25	220.53	169.43	282.36	−0.07	−0.35	0.34
Qom	Males	367.47	276.03	484.11	325.16	251.69	413.66	−0.12	−0.39	0.27
Semnan	Males	289.04	219.31	384.44	192.44	148.03	256.73	−0.33	−0.53	−0.03
Sistan and Baluchistan	Males	224.44	160.99	362.99	294.01	223.81	421.41	0.31	−0.12	0.88
South Khorasan	Males	236.57	173.87	343.12	221.15	178.26	280.61	−0.07	−0.33	0.34
Tehran	Males	252.38	184.91	333.36	163.33	127.01	219.3	−0.35	−0.54	−0.03
West Azarbayejan	Males	509.17	372.31	662.57	408.53	289.07	502.87	−0.2	−0.43	0.14
Yazd	Males	221.89	164.94	358.27	173.3	129.73	239.73	−0.22	−0.45	0.14
Zanjan	Males	253.86	193.33	330.9	184.33	144.28	242.46	−0.27	−0.48	0.06
Iran (Islamic Republic of)	Females	303.11	202.92	343.37	124.44	108.73	155.18	−0.59	−0.66	−0.42
Alborz	Females	273.44	153.68	390.8	139.71	98.2	182.74	−0.49	−0.68	−0.08
Ardebil	Females	380.15	226.79	510.12	146.79	106.84	178.62	−0.61	−0.72	−0.4
Bushehr	Females	426.38	194.12	599.11	155.23	97.02	193.26	−0.64	−0.75	−0.42
Chahar Mahaal and Bakhtiari	Females	425.11	178.38	591.25	132.98	71.17	182.46	−0.69	−0.8	−0.49
East Azarbayejan	Females	298.01	190.39	411.07	124.41	93.01	167.63	−0.58	−0.73	−0.32
Fars	Females	385.15	210.57	522.91	178.03	132.2	233.03	−0.54	−0.68	−0.2
Gilan	Females	276.55	171.73	378.93	110.85	84.48	144.12	−0.6	−0.74	−0.36
Golestan	Females	601.83	227.7	837.06	226.11	121.39	296.84	−0.62	−0.75	−0.42
Hamadan	Females	364.15	203.55	488.33	154.19	112.64	196.73	−0.58	−0.71	−0.34
Hormozgan	Females	262.49	178.8	363.78	85.31	64.19	140.85	−0.67	−0.78	−0.42
Ilam	Females	954.67	239.66	1,313.03	338.08	122.5	432.97	−0.65	−0.75	−0.38
Isfahan	Females	126.42	89.13	223.27	59.08	41.49	128.59	−0.53	−0.69	−0.29
Kerman	Females	321.43	218.47	460.37	137.46	101.5	206.1	−0.57	−0.73	−0.31
Kermanshah	Females	733.54	273.89	1,038.41	307.61	146.28	414.09	−0.58	−0.71	−0.34
Khorasan-e-Razavi	Females	328.9	211.97	445.44	114.95	87.06	160.73	−0.65	−0.77	−0.39
Khuzestan	Females	364.66	243.34	504.77	171.33	129.64	219.08	−0.53	−0.68	−0.28
Kohgiluyeh and Boyer-Ahmad	Females	594.83	226.95	820.87	323.95	175.16	419.69	−0.46	−0.62	−0.17
Kurdistan	Females	445.34	228.6	604.68	131.16	91.25	167.77	−0.71	−0.8	−0.52
Lorestan	Females	740.93	186.48	1,033.89	243.63	90.3	318.12	−0.67	−0.77	−0.43
Markazi	Females	169.85	115.91	290.92	59.69	42.24	141.3	−0.65	−0.77	−0.46
Mazandaran	Females	182.45	126.12	267.71	90.27	67.65	148.97	−0.51	−0.69	−0.21
North Khorasan	Females	437.35	262.32	584.12	196.46	142.49	252.85	−0.55	−0.7	−0.27
Qazvin	Females	207.87	150.08	286.34	75.01	53.73	111.44	−0.64	−0.76	−0.39
Qom	Females	86.97	53.75	229.17	31.34	19.87	107.53	−0.64	−0.77	−0.47
Semnan	Females	139.16	99.41	210.76	46.91	32.94	95.89	−0.66	−0.78	−0.45
Sistan and Baluchistan	Females	152.18	100.86	227.23	100.09	68.32	256.9	−0.34	−0.61	0.27
South Khorasan	Females	209.04	140.87	286.51	92.16	72.32	139.86	−0.56	−0.68	−0.32
Tehran	Females	83.99	52.92	187.24	49.94	34.47	118.61	−0.41	−0.62	−0.07
West Azarbayejan	Females	568.94	298.05	785.6	257.42	173.48	326.7	−0.55	−0.69	−0.34
Yazd	Females	177.79	129.8	246.98	65.08	46.07	111.03	−0.63	−0.76	−0.44
Zanjan	Females	212.63	148.03	290.36	74.54	56.97	111.17	−0.65	−0.77	−0.4
All-ages DALYs counts estimates										
Iran (Islamic Republic of)	Males	105,109.67	88,800.06	118,654.48	150,642.85	129,386.56	165,530.23	0.43	0.25	0.68
Alborz	Males	4,785.14	1,977.87	6,546.54	9,620.22	4,583.93	12,427.56	1.01	0.39	2.16
Ardebil	Males	2,238.31	1,566.58	2,962.58	3,140.48	2,135.42	3,791.58	0.4	0.02	0.95
Bushehr	Males	792.73	581.01	1,105.82	1,353.29	1,056.52	1,733.32	0.71	0.21	1.43
Chahar Mahaal and Bakhtiari	Males	1,168.91	852.52	1,513.74	1,278.57	1,001.65	1,637.58	0.09	−0.23	0.65
East Azarbayejan	Males	8,819.41	5,859.44	11,866.60	8,886.08	6,519.33	11,162.29	0.01	−0.29	0.44
Fars	Males	7,240.32	5,363.35	9,567.00	12,688.20	9,659.50	16,177.91	0.75	0.22	1.47
Gilan	Males	4,827.20	3,470.78	6,426.42	5,302.00	3,935.33	6,774.20	0.1	−0.22	0.55
Golestan	Males	2,158.03	1,596.81	2,788.46	3,929.64	3,140.74	4,904.34	0.82	0.33	1.48
Hamadan	Males	5,909.33	3,462.24	7,788.94	6,295.59	3,462.19	7,975.75	0.07	−0.26	0.44
Hormozgan	Males	1,757.61	1,257.63	2,384.00	4,619.52	3,488.14	5,866.08	1.63	0.81	2.94
Ilam	Males	1,562.63	656.45	2,106.58	2,700.39	1,275.84	3,377.95	0.73	0.23	1.41
Isfahan	Males	4,300.99	3,121.74	6,405.25	6,536.09	5,108.41	8,657.84	0.52	0.05	1.26
Kerman	Males	3,543.70	2,688.95	4,708.34	6,014.05	4,802.83	7,730.71	0.7	0.2	1.44
Kermanshah	Males	6,617.79	4,107.53	8,759.14	7,130.22	4,071.72	8,845.19	0.08	−0.24	0.5
Khorasan-e-Razavi	Males	7,922.77	5,805.93	10,472.49	10,315.00	8,102.14	12,809.88	0.3	−0.09	0.84
Khuzestan	Males	4,573.24	3,421.02	6,045.35	8,760.33	7,013.53	10,703.06	0.92	0.39	1.68
Kohgiluyeh and Boyer-Ahmad	Males	1,283.36	875.53	1,714.53	2,415.38	1,762.24	3,025.18	0.88	0.31	1.76
Kurdistan	Males	2,213.84	1,575.17	2,953.90	3,032.71	2,365.45	3,793.67	0.37	−0.05	1.06
Lorestan	Males	4,735.00	2,375.03	6,287.15	5,044.98	2,681.32	6,399.49	0.07	−0.24	0.53
Markazi	Males	2,384.36	1,755.07	3,084.16	2,283.17	1,785.47	2,874.05	−0.04	−0.32	0.36
Mazandaran	Males	2,627.34	1,935.90	3,921.82	4,261.76	3,402.84	5,691.96	0.62	0.15	1.37
North Khorasan	Males	887.39	663.14	1,252.67	1,290.11	1,028.98	1,638.99	0.45	0.04	1.06
Qazvin	Males	988.27	736.08	1,434.88	1,677.70	1,287.92	2,131.81	0.7	0.2	1.45
Qom	Males	1,269.80	952.6	1,663.77	2,540.10	1,979.52	3,210.06	1	0.4	1.87
Semnan	Males	643.45	482.9	858.41	867.68	669.82	1,149.98	0.35	−0.04	0.99
Sistan and Baluchistan	Males	1,350.61	950.06	2,021.48	4,677.26	3,526.83	6,687.79	2.46	1.35	4.16
South Khorasan	Males	665.21	479.13	944.35	1,054.71	848.67	1,327.53	0.59	0.14	1.27
Tehran	Males	10,485.67	7,640.32	13,685.69	12,997.11	10,090.36	17,426.77	0.24	−0.13	0.84
West Azarbayejan	Males	5,702.28	4,097.11	7,618.92	7,695.77	5,446.80	9,502.46	0.35	−0.05	0.95
Yazd	Males	691.59	505.54	1,090.36	1,107.88	837.76	1,536.14	0.6	0.1	1.34
Zanjan	Males	963.33	716.54	1,284.21	1,126.86	886.07	1,474.55	0.17	−0.18	0.72
Iran (Islamic Republic of)	Females	83,677.70	53,304.15	95,584.67	52,614.69	45,850.79	66,073.28	−0.37	−0.49	−0.07
Alborz	Females	1,902.17	1,030.34	2,777.59	2,074.58	1,468.43	2,719.96	0.09	−0.31	1.03
Ardebil	Females	2,060.16	1,172.19	2,818.16	955.16	695.95	1,171.13	−0.54	−0.68	−0.27
Bushehr	Females	1,433.61	634.1	2,033.64	989.4	623.31	1,229.33	−0.31	−0.53	0.12
Chahar Mahaal and Bakhtiari	Females	1,403.85	548.8	1,991.43	680.1	367.91	918.53	−0.52	−0.69	−0.19
East Azarbayejan	Females	5,021.42	3,137.44	6,997.12	2,415.13	1,825.18	3,279.25	−0.52	−0.68	−0.19
Fars	Females	6,614.35	3,453.55	9,141.91	4,494.10	3,334.20	5,960.97	−0.32	−0.54	0.26
Gilan	Females	3,159.35	1,882.83	4,445.75	1,442.61	1,100.67	1,878.44	−0.54	−0.71	−0.25
Golestan	Females	3,865.98	1,413.45	5,434.44	2,256.45	1,225.93	2,975.43	−0.42	−0.62	−0.06
Hamadan	Females	2,931.52	1,608.62	3,991.56	1,283.19	963.08	1,618.84	−0.56	−0.7	−0.3
Hormozgan	Females	1,111.61	736.35	1,567.15	858.09	644.95	1,432.46	−0.23	−0.5	0.43
Ilam	Females	1,934.65	456.1	2,695.96	1,107.88	403.16	1,441.32	−0.43	−0.6	0.03
Isfahan	Females	2,300.84	1,605.67	3,919.77	1,558.55	1,098.17	3,480.22	−0.32	−0.56	0.04
Kerman	Females	2,756.65	1,823.94	4,029.96	2,290.01	1,702.28	3,449.73	−0.17	−0.48	0.37
Kermanshah	Females	5,899.59	2,165.86	8,473.20	3,026.95	1,455.77	4,065.35	−0.49	−0.65	−0.14
Khorasan-e-Razavi	Females	7,289.02	4,593.40	9,929.28	3,928.37	2,964.65	5,520.13	−0.46	−0.65	−0.01
Khuzestan	Females	5,332.78	3,422.79	7,422.09	4,189.12	3,217.65	5,371.54	−0.21	−0.48	0.22
Kohgiluyeh and Boyer-Ahmad	Females	1,418.10	520.69	1,949.37	1,251.51	698.94	1,614.59	−0.12	−0.41	0.46
Kurdistan	Females	2,671.71	1,306.05	3,624.85	1,156.29	829.01	1,478.96	−0.57	−0.7	−0.28
Lorestan	Females	5,397.03	1,277.35	7,642.97	2,246.37	850.28	2,932.28	−0.58	−0.71	−0.24
Markazi	Females	991.16	668.58	1,656.46	433.62	309.85	1,035.31	−0.56	−0.71	−0.3
Mazandaran	Females	2,368.52	1,587.49	3,453.79	1,567.71	1,176.28	2,641.40	−0.34	−0.59	0.06
North Khorasan	Females	1,340.82	771.66	1,829.43	833.94	613.13	1,061.11	−0.38	−0.58	0.02
Qazvin	Females	932.49	651.71	1,276.84	516.13	369.8	773.08	−0.45	−0.64	−0.06
Qom	Females	296.17	177.94	780.29	228.53	144.94	791.67	−0.23	−0.53	0.17
Semnan	Females	314.76	220.79	462.81	194.31	136.6	401.39	−0.38	−0.61	0.03
Sistan and Baluchistan	Females	979.05	613.79	1,443.46	1,556.90	1,054.02	4,003.73	0.59	−0.09	2.55
South Khorasan	Females	631.96	423.94	864.7	398.22	312.79	610.75	−0.37	−0.54	0
Tehran	Females	3,351.44	2,067.81	7,478.63	3,564.46	2,481.47	8,516.25	0.06	−0.33	0.74
West Azarbayejan	Females	6,546.79	3,219.58	9,091.93	4,320.23	3,012.45	5,442.08	−0.34	−0.56	−0.01
Yazd	Females	539.19	392.81	755.47	385.07	271.44	662.05	−0.29	−0.55	0.11
Zanjan	Females	880.97	582.36	1,226.49	411.7	316.38	625.77	−0.53	−0.7	−0.17

**Figure 6.**
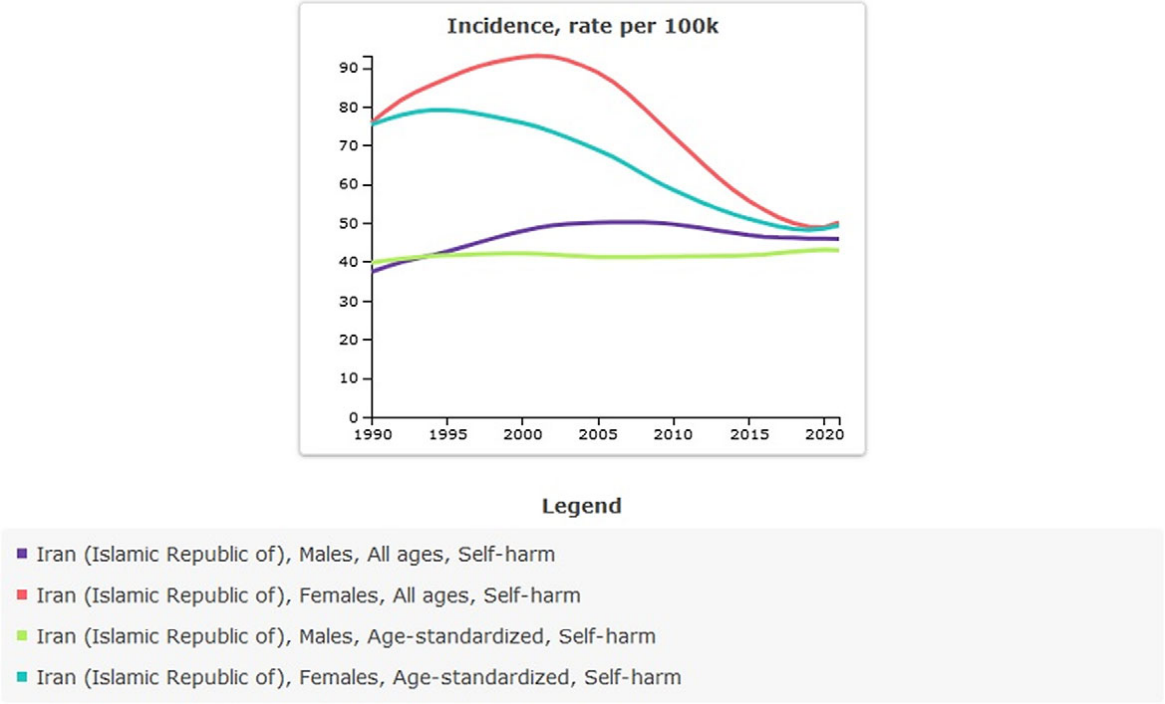
Sex-specific incidence of self-harm in Iran, 1990–2021.

**Figure 7.**
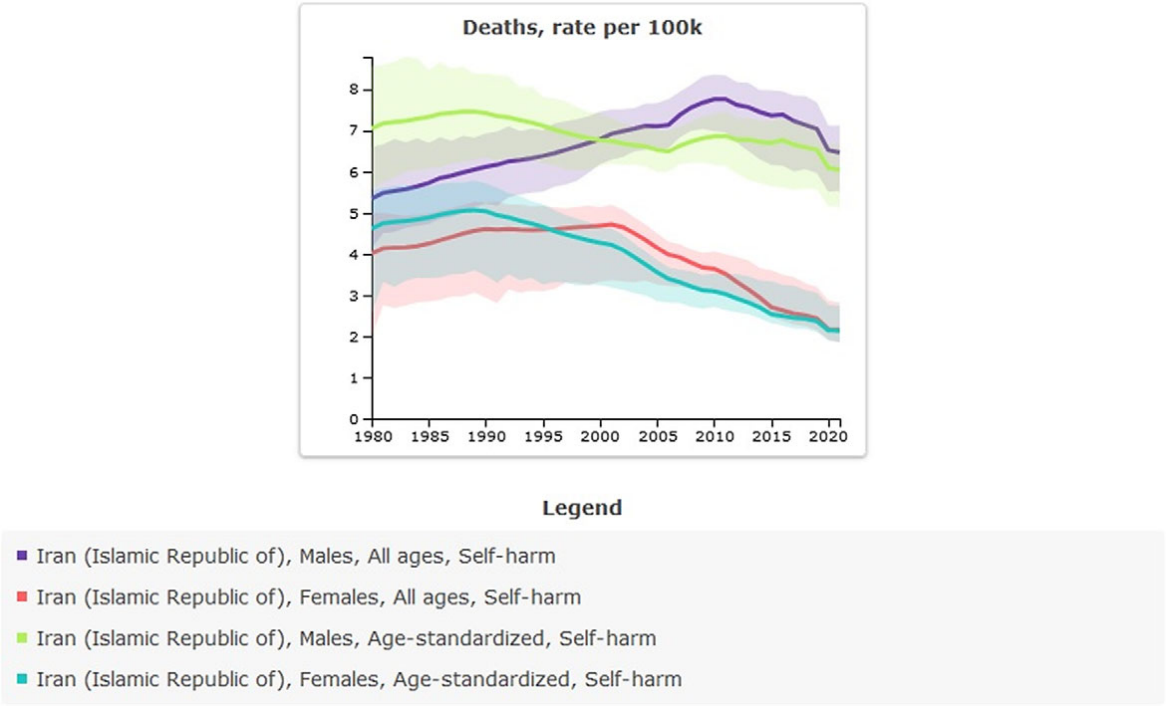
Sex-specific suicide mortality in Iran, 1990–2021.

The incidence of self-harm in males and females has had a different trend. Since 1990, the incidence of self-harm in males has shown a relatively stable trend with a slight increase. In contrast, a decreasing trend can be seen in females; however, in 2020, the incidence was close to that of males, and in 2021, it shows an increasing trend again (Table 5 and Figure 6). In 2021, age-standardized rate of suicide mortality (per 100,000) in males was 6.04 (95% UI: 5.14–6.67), and in females was 2.14 (95% UI: 1.85–2.74). The suicide mortality rate in males compared to females has been higher since 1990 and has increased over time. In 2021, there were 2,398 suicide mortality among males, compared with 906 suicide mortality among females (Table 6 and Figure 7).

**Table 6.** Sex-specific suicide mortality in Iran, stratified by provinces, 1990–2021

Location	Sex	Year								
		1990			2021			Percentage change 1990–2021		
		Value	Lower	Upper	Value	Lower	Upper	Value	Lower	Upper
Age-standardized rate suicide mortality (per 100,000)										
Iran (Islamic Republic of)	Males	7.42	6.35	8.39	6.04	5.14	6.67	−0.19	−0.29	−0.03
Alborz	Males	13.42	5.54	18.33	10.67	4.94	13.88	−0.21	−0.44	0.26
Ardebil	Males	8.26	6.12	10.58	8.33	5.52	9.96	0.01	−0.28	0.41
Bushehr	Males	4.94	3.66	7.21	3.69	2.9	4.71	−0.25	−0.47	0.04
Chahar Mahaal and Bakhtiari	Males	7.3	5.36	9.34	4.57	3.6	5.77	−0.37	−0.55	−0.05
East Azarbayejan	Males	9.76	6.97	12.94	7.57	5.48	9.5	−0.22	−0.45	0.11
Fars	Males	7.92	5.98	10.55	8.09	6.11	10.31	0.02	−0.29	0.46
Gilan	Males	8.66	6.43	11.48	7.06	5.2	8.98	−0.18	−0.42	0.15
Golestan	Males	7.09	5.29	9.49	7.21	5.75	9.1	0.02	−0.26	0.4
Hamadan	Males	13.75	8.37	18.23	12.31	6.64	15.66	−0.1	−0.38	0.22
Hormozgan	Males	8.47	6.18	11.37	7.68	5.8	9.75	−0.09	−0.36	0.32
Ilam	Males	15.56	6.84	20.51	14.67	6.6	18.34	−0.06	−0.32	0.34
Isfahan	Males	4.12	3.01	6.56	4.12	3.21	5.4	0	−0.32	0.5
Kerman	Males	8.09	6.17	10.97	5.93	4.69	7.67	−0.27	−0.48	0.07
Kermanshah	Males	15.59	10.24	20.15	11.89	6.73	14.85	−0.24	−0.46	0.06
Khorasan-e-Razavi	Males	7.28	5.46	9.64	5.34	4.18	6.68	−0.27	−0.49	0.05
Khuzestan	Males	6.13	4.62	8.38	6.09	4.89	7.4	−0.01	−0.28	0.39
Kohgiluyeh and Boyer-Ahmad	Males	9.57	6.86	12.39	9.35	6.73	11.6	−0.02	−0.31	0.39
Kurdistan	Males	7.49	5.36	9.98	5.77	4.52	7.28	−0.23	−0.47	0.16
Lorestan	Males	13.56	7.25	17.69	10.1	5.3	12.58	−0.26	−0.46	0.05
Markazi	Males	7.74	5.72	10.18	5.22	4.1	6.63	−0.33	−0.52	−0.03
Mazandaran	Males	3.94	2.9	6.3	4.15	3.3	5.64	0.05	−0.26	0.53
North Khorasan	Males	5.79	4.34	8.22	5.16	4.14	6.48	−0.11	−0.36	0.3
Qazvin	Males	4.63	3.48	7.16	4.3	3.35	5.47	−0.07	−0.35	0.33
Qom	Males	6.96	5.26	9.26	5.96	4.6	7.54	−0.14	−0.42	0.21
Semnan	Males	5.59	4.21	7.53	3.71	2.87	4.89	−0.34	−0.53	−0.03
Sistan and Baluchistan	Males	4.22	3.02	7.45	5.28	4	7.7	0.25	−0.15	0.82
South Khorasan	Males	4.45	3.28	6.51	4.13	3.32	5.23	−0.07	−0.35	0.35
Tehran	Males	4.76	3.48	6.41	3.11	2.4	4.14	−0.35	−0.54	−0.01
West Azarbayejan	Males	9.49	6.91	12.27	7.7	5.45	9.44	−0.19	−0.42	0.15
Yazd	Males	4.28	3.17	7.09	3.22	2.39	4.49	−0.25	−0.47	0.09
Zanjan	Males	4.89	3.72	6.53	3.6	2.85	4.76	−0.26	−0.47	0.06
Iran (Islamic Republic of)	Females	5.04	3.47	5.71	2.14	1.85	2.74	−0.58	−0.65	−0.4
Alborz	Females	4.71	2.58	6.69	2.49	1.74	3.27	−0.47	−0.66	0.01
Ardebil	Females	6.29	3.65	8.47	2.59	1.88	3.2	−0.59	−0.71	−0.35
Bushehr	Females	7.35	3.15	10.38	2.86	1.73	3.55	−0.61	−0.73	−0.37
Chahar Mahaal and Bakhtiari	Females	7.21	2.78	9.91	2.38	1.18	3.23	−0.67	−0.78	−0.45
East Azarbayejan	Females	4.89	3.13	6.83	2.1	1.55	2.87	−0.57	−0.72	−0.27
Fars	Females	6.45	3.53	8.82	3.02	2.22	4.01	−0.53	−0.69	−0.19
Gilan	Females	4.78	2.98	6.55	1.99	1.48	2.61	−0.58	−0.73	−0.33
Golestan	Females	10.02	3.54	13.97	4.1	2.09	5.35	−0.59	−0.72	−0.36
Hamadan	Females	6.03	3.22	8.16	2.61	1.93	3.33	−0.57	−0.7	−0.31
Hormozgan	Females	4.3	2.84	6.02	1.48	1.08	2.62	−0.66	−0.77	−0.38
Ilam	Females	16.74	3.82	23.06	6.54	2.21	8.38	−0.61	−0.72	−0.29
Isfahan	Females	2.05	1.4	3.89	0.98	0.66	2.34	−0.52	−0.69	−0.27
Kerman	Females	5.36	3.61	7.65	2.33	1.71	3.69	−0.57	−0.73	−0.29
Kermanshah	Females	12.39	4.33	17.53	5.33	2.43	7.17	−0.57	−0.71	−0.3
Khorasan-e-Razavi	Females	5.44	3.49	7.41	1.98	1.5	2.77	−0.64	−0.76	−0.36
Khuzestan	Females	6.12	4.09	8.5	2.94	2.27	3.78	−0.52	−0.67	−0.25
Kohgiluyeh and Boyer-Ahmad	Females	9.77	3.55	13.42	5.37	2.89	6.96	−0.45	−0.61	−0.13
Kurdistan	Females	7.28	3.58	10.06	2.34	1.61	3.04	−0.68	−0.78	−0.47
Lorestan	Females	12.72	2.92	17.86	4.46	1.48	5.85	−0.65	−0.76	−0.39
Markazi	Females	2.74	1.84	5.02	0.98	0.68	2.56	−0.64	−0.77	−0.43
Mazandaran	Females	3.05	2.07	4.58	1.55	1.14	2.7	−0.49	−0.68	−0.19
North Khorasan	Females	7.22	4.35	9.72	3.39	2.48	4.36	−0.53	−0.68	−0.2
Qazvin	Females	3.48	2.51	4.82	1.3	0.93	2.01	−0.63	−0.76	−0.35
Qom	Females	1.41	0.83	4.09	0.52	0.31	2.05	−0.63	−0.78	−0.44
Semnan	Females	2.28	1.59	3.71	0.78	0.53	1.72	−0.66	−0.79	−0.42
Sistan and Baluchistan	Females	2.47	1.58	4.04	1.61	1.06	4.46	−0.35	−0.62	0.2
South Khorasan	Females	3.48	2.36	4.77	1.58	1.22	2.45	−0.55	−0.69	−0.3
Tehran	Females	1.37	0.81	3.29	0.82	0.55	2.1	−0.4	−0.63	−0.07
West Azarbayejan	Females	9.46	4.98	13.12	4.38	3	5.59	−0.54	−0.68	−0.32
Yazd	Females	2.98	2.12	4.2	1.1	0.77	1.99	−0.63	−0.76	−0.43
Zanjan	Females	3.55	2.46	4.77	1.3	0.98	2.03	−0.63	−0.76	−0.38
All-ages counts estimates										
Iran (Islamic Republic of)	Males	1,779.40	1,522.33	2,017.04	2,802.33	2,398.13	3,090.06	0.57	0.38	0.87
Alborz	Males	82.52	33.68	112.88	181.11	84.22	235.1	1.19	0.52	2.43
Ardebil	Males	37.7	26.98	49.02	60.47	40.73	73.02	0.6	0.16	1.24
Bushehr	Males	13.5	9.83	19.07	25.43	19.86	32.9	0.88	0.36	1.67
Chahar Mahaal and Bakhtiari	Males	20.06	14.73	25.74	24.59	19.31	31.53	0.23	−0.13	0.85
East Azarbayejan	Males	146.65	99.22	196.33	169.72	123.17	213.62	0.16	−0.18	0.68
Fars	Males	120.67	90.2	159.89	228.59	173.45	290.49	0.89	0.33	1.68
Gilan	Males	84.84	61.25	113.09	104.35	76.56	132.17	0.23	−0.13	0.73
Golestan	Males	36.67	27.04	48.18	72.12	57.49	90.58	0.97	0.43	1.7
Hamadan	Males	99.5	59	131.23	119.22	64.24	151.64	0.2	−0.17	0.64
Hormozgan	Males	30.28	21.58	40.85	80.55	60.46	101.58	1.66	0.85	3.02
Ilam	Males	26.7	11.28	35.64	49.62	22.89	62.18	0.86	0.33	1.62
Isfahan	Males	70.69	51.48	108.4	121.2	94.64	159.93	0.71	0.17	1.53
Kerman	Males	60	45.49	80.58	108.66	85.91	140.07	0.81	0.27	1.64
Kermanshah	Males	111.58	70.41	146.73	133.1	75.68	165.75	0.19	−0.15	0.68
Khorasan-e-Razavi	Males	136.15	100.55	182.87	193.01	150.75	239.63	0.42	−0.01	1
Khuzestan	Males	76.57	57.33	103.55	158.01	127.39	191.2	1.06	0.5	1.89
Kohgiluyeh and Boyer-Ahmad	Males	20.23	14.03	26.55	41.58	30.32	52	1.06	0.44	2
Kurdistan	Males	37.57	26.45	50.24	55.57	43.37	70.26	0.48	0.01	1.25
Lorestan	Males	81.28	41.66	107.26	97.83	51.14	123.76	0.2	−0.15	0.71
Markazi	Males	39.89	29.49	51.65	42.59	33.34	54.4	0.07	−0.24	0.52
Mazandaran	Males	43.78	31.84	67.74	80.84	64.1	108.27	0.85	0.31	1.71
North Khorasan	Males	14.97	11.18	20.68	23.6	18.8	29.88	0.58	0.11	1.26
Qazvin	Males	17.05	12.75	25.37	32.35	24.8	41.1	0.9	0.34	1.74
Qom	Males	21.34	16.02	28.15	46.05	35.76	58.16	1.16	0.49	2.16
Semnan	Males	11.33	8.53	15.14	16.38	12.63	21.61	0.45	0.03	1.14
Sistan and Baluchistan	Males	22.87	16.19	36.01	79.56	59.88	114.86	2.48	1.31	4.18
South Khorasan	Males	11.56	8.39	16.9	19.29	15.55	24.44	0.67	0.18	1.39
Tehran	Males	180.39	130.55	240.71	252.6	194.38	338.41	0.4	−0.04	1.09
West Azarbayejan	Males	94.39	68.78	124.41	142.16	100.73	176.06	0.51	0.06	1.17
Yazd	Males	11.97	8.82	19.34	20.3	15.31	28.42	0.7	0.17	1.48
Zanjan	Males	16.7	12.55	22.13	21.85	17.21	28.7	0.31	−0.07	0.89
Iran (Islamic Republic of)	Females	1,290.26	820.94	1,468.85	906.23	779.55	1,182.62	−0.3	−0.43	0.04
Alborz	Females	29.73	15.9	43.27	35.98	24.9	48.23	0.21	−0.26	1.39
Ardebil	Females	31.41	17.52	42.92	17.14	12.48	21.23	−0.45	−0.62	−0.12
Bushehr	Females	22.43	9.57	32.18	17.49	10.71	21.9	−0.22	−0.47	0.3
Chahar Mahaal and Bakhtiari	Females	21.77	8.15	31.02	12.2	6.09	16.57	−0.44	−0.64	−0.03
East Azarbayejan	Females	76.23	46.51	106.41	40.97	30.08	57.76	−0.46	−0.66	−0.06
Fars	Females	102.51	52.84	141.62	76.9	56.31	101.42	−0.25	−0.5	0.42
Gilan	Females	50.56	30.4	70.31	27.02	20.1	35.61	−0.47	−0.67	−0.1
Golestan	Females	59.63	20.74	83.94	40.82	21.09	53.83	−0.32	−0.55	0.11
Hamadan	Females	45.16	24.1	61.99	22.37	16.4	28.08	−0.5	−0.67	−0.19
Hormozgan	Females	16.96	11.04	24	14.27	10.33	25.47	−0.16	−0.46	0.58
Ilam	Females	30.44	6.76	42.42	21.16	7.34	27.69	−0.3	−0.51	0.27
Isfahan	Females	34.73	23.73	63.22	25.92	17.38	64.09	−0.25	−0.54	0.17
Kerman	Females	42.64	28.13	62.27	38.15	27.94	61.45	−0.11	−0.45	0.48
Kermanshah	Females	91.75	32.05	132.64	53.57	24.88	71.51	−0.42	−0.61	0
Khorasan-e-Razavi	Females	111.84	70.05	153.61	66.83	50.25	94.77	−0.4	−0.61	0.1
Khuzestan	Females	82.8	52.89	117.11	70.88	54.94	92.86	−0.14	−0.43	0.35
Kohgiluyeh and Boyer-Ahmad	Females	21.36	7.56	29.49	20.67	11.29	27	−0.03	−0.35	0.6
Kurdistan	Females	40.52	19.06	55.57	20.66	14.58	26.79	−0.49	−0.66	−0.11
Lorestan	Females	84.45	18.4	119.71	41.3	13.97	54.39	−0.51	−0.67	−0.12
Markazi	Females	14.98	10.03	26.49	7.33	5.06	19.61	−0.51	−0.69	−0.19
Mazandaran	Females	36.63	24.02	54.47	27.81	20.4	49.95	−0.24	−0.54	0.22
North Khorasan	Females	20.54	11.74	27.95	14.32	10.5	18.6	−0.3	−0.54	0.17
Qazvin	Females	14.41	10.14	19.95	8.94	6.4	14.01	−0.38	−0.61	0.1
Qom	Females	4.41	2.54	12.46	3.61	2.12	14.4	−0.18	−0.54	0.32
Semnan	Females	4.88	3.34	7.71	3.22	2.15	7.12	−0.34	−0.61	0.14
Sistan and Baluchistan	Females	14.75	9.32	22.19	23.98	15.76	65.51	0.62	−0.09	2.53
South Khorasan	Females	9.77	6.52	13.48	6.79	5.27	10.69	−0.3	−0.52	0.1
Tehran	Females	50.82	29.55	122.56	59.5	38.87	155.34	0.17	−0.3	0.96
West Azarbayejan	Females	100.16	49.29	139.81	72.76	50.63	92.15	−0.27	−0.51	0.12
Yazd	Females	8.42	5.98	11.93	6.39	4.42	11.53	−0.24	−0.53	0.21
Zanjan	Females	13.57	9.2	18.95	7.28	5.45	11.52	−0.46	−0.65	−0.05

### The age-specific burden of self-harm and suicide mortality in Iran

**Figure 8.**
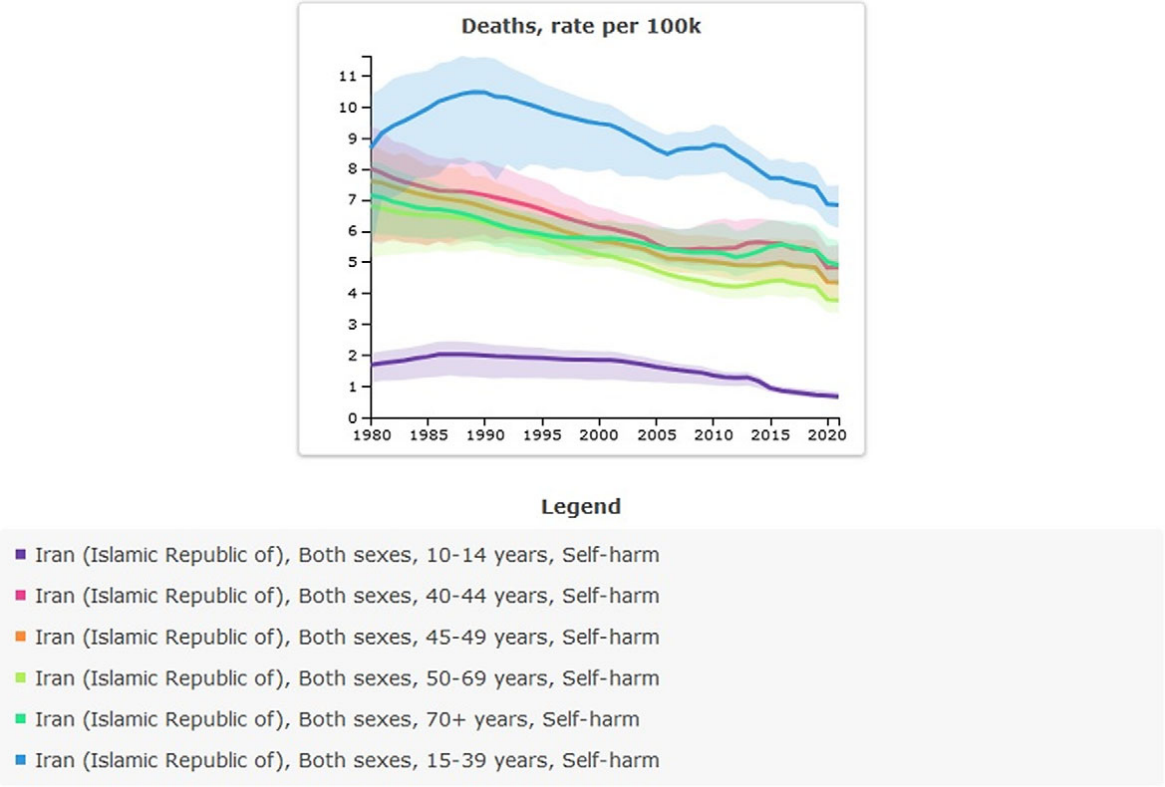
Age-specific suicide mortality in Iran, 1990–2021.

In 2021, the age-specific rate of suicide mortality (per 100,000) was highest among individuals aged 15–39 years, at 6.82 (95% UI: 6.09–7.48). For both males and females, the highest age-specific rate of suicide mortality (per 100,000) was among individuals aged 15–39 years, at 10.01 (95% UI: 8.57–11.06) and 3.51 (95% UI: 3–4.42), respectively (Table 7 and Figure 8).

**Table 7.** Age-specific suicide mortality in Iran, stratified by provinces, 1990–2021

Age	Sex	Year								
		1990			2021			Percentage change 1990–2021		
		Value	Lower	Upper	Value	Lower	Upper	Value	Lower	Upper
Age− rate suicide mortality (per 100,000)										
10−14 years	Both sexes	1.99	1.26	2.36	0.66	0.55	0.81	−0.67	−0.74	−0.51
15−39 years	Both sexes	10.46	8.07	11.6	6.82	6.09	7.48	−0.35	−0.43	−0.19
40−44 years	Both sexes	7.15	5.87	8.26	4.83	4.3	5.55	−0.32	−0.42	−0.1
45−49 years	Both sexes	6.76	5.65	7.76	4.34	3.85	5.09	−0.36	−0.45	−0.13
50−69 years	Both sexes	6.26	5.39	6.99	3.77	3.37	4.38	−0.4	−0.46	−0.23
70+ years	Both sexes	6.35	5.63	7.09	4.89	4.26	5.67	−0.23	−0.31	−0.06
10−14 years	Males	2.02	1.26	2.49	0.71	0.55	0.94	−0.65	−0.74	−0.46
15−39 years	Males	11.28	9.44	12.95	10.01	8.57	11.06	−0.11	−0.24	0.05
40−44 years	Males	9.41	7.51	11.14	7.53	6.37	8.65	−0.2	−0.34	0.06
45−49 years	Males	9.36	7.73	11.13	6.71	5.73	7.88	−0.28	−0.41	−0.01
50−69 years	Males	8.87	7.57	10.02	5.96	5.08	6.83	−0.33	−0.42	−0.14
70+ years	Males	8	7.02	9.41	6.81	5.8	7.86	−0.15	−0.27	0.04
10−14 years	Females	1.96	1.03	2.44	0.61	0.47	0.76	−0.69	−0.76	−0.52
15−39 years	Females	9.61	5.92	11.14	3.51	3	4.42	−0.63	−0.72	−0.44
40−44 years	Females	4.84	3.55	5.61	2.05	1.71	2.79	−0.58	−0.66	−0.36
45−49 years	Females	4.1	3.01	5.02	1.85	1.56	2.78	−0.55	−0.64	−0.33
50−69 years	Females	3.25	2.49	3.77	1.58	1.34	2.29	−0.52	−0.6	−0.33
70+ years	Females	4.64	3.61	5.63	3.03	2.54	4.09	−0.35	−0.45	−0.18
Age count estimates										
10−14 years	Both sexes	144.58	91.89	171.23	43.7	36.68	53.76	−0.7	−0.76	−0.55
15−39 years	Both sexes	2,270.81	1,752.85	2,519.34	2,367.98	2,112.17	2,594.32	0.04	−0.09	0.3
40−44 years	Both sexes	155.59	127.84	179.74	344.75	307.21	396.52	1.22	0.91	1.95
45−49 years	Both sexes	110.47	92.3	126.89	240.73	213.44	282.62	1.18	0.87	1.95
50−69 years	Both sexes	326.58	280.94	364.8	532.74	475.98	618.61	0.63	0.46	1.09
70+ years	Both sexes	61.63	54.63	68.8	178.66	155.69	207.09	1.9	1.61	2.52
10−14 years	Males	74.96	46.65	92.31	24.02	18.61	31.84	−0.68	−0.76	−0.51
15−39 years	Males	1,236.31	1,034.02	1,418.77	1,770.98	1,515.40	1,957.52	0.43	0.23	0.69
40−44 years	Males	103.3	82.38	122.23	272.67	230.54	313.32	1.64	1.17	2.49
45−49 years	Males	77.35	63.89	92.02	190.51	162.71	223.73	1.46	1.04	2.41
50−69 years	Males	247.96	211.57	280.17	421.68	359.75	483.25	0.7	0.46	1.18
70+ years	Males	39.51	34.7	46.47	122.46	104.41	141.45	2.1	1.65	2.8
10−14 years	Females	69.62	36.64	86.84	19.68	15.19	24.7	−0.72	−0.79	−0.57
15−39 years	Females	1,034.50	636.5	1,199.05	596.99	509.97	751.49	−0.42	−0.55	−0.12
40−44 years	Females	52.29	38.33	60.57	72.08	60.19	98.39	0.38	0.1	1.07
45−49 years	Females	33.12	24.32	40.59	50.22	42.21	75.52	0.52	0.19	1.25
50−69 years	Females	78.62	60.26	91.32	111.05	94.64	161.64	0.41	0.16	0.94
70+ years	Females	22.11	17.21	26.82	56.2	47.14	75.68	1.54	1.13	2.19

### The burden of self-harm and suicide mortality stratified by provinces

**Figure 9.**
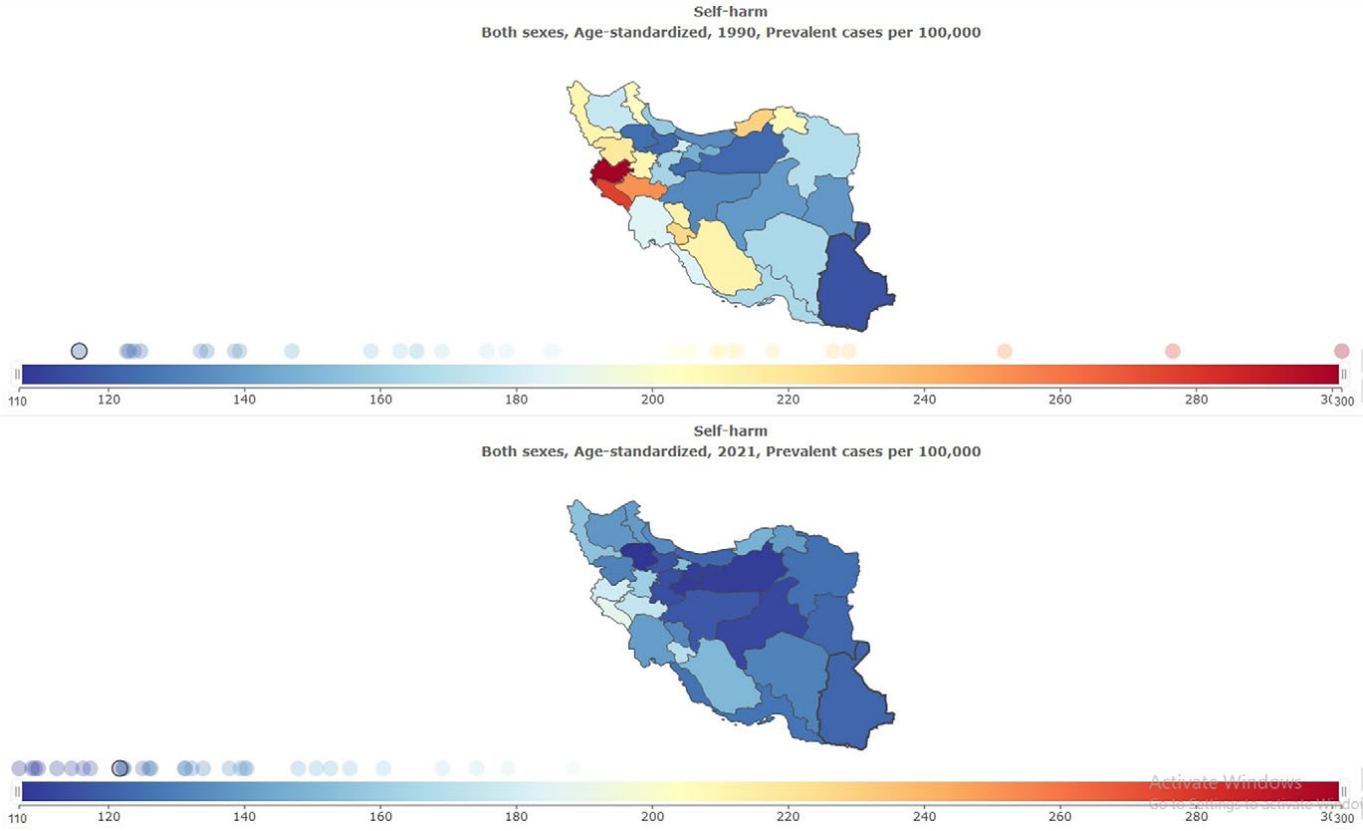
Age-standardized rate (per 100,000) prevalence of self-harm in Iran, stratified by provinces, 1990–2021.

**Figure 10.**
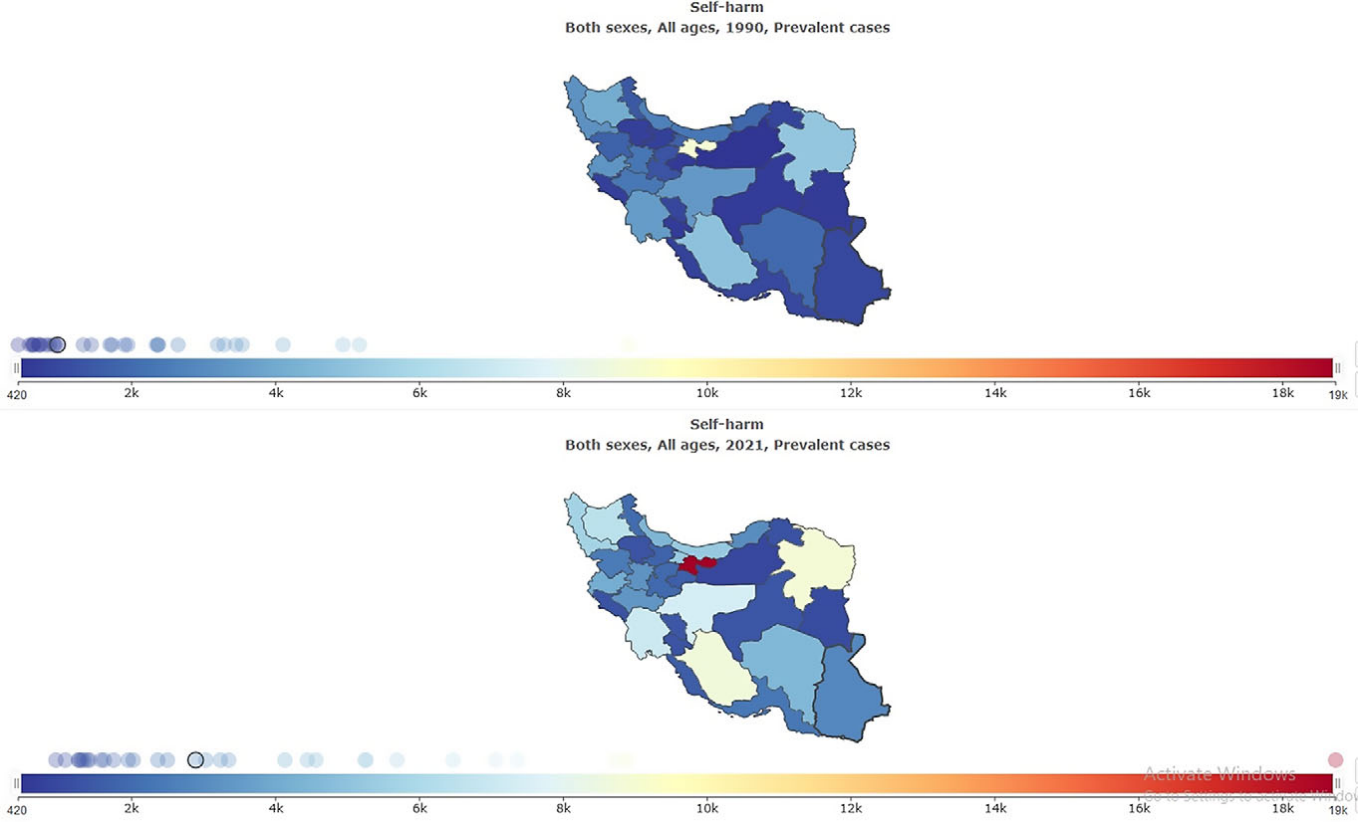
All-ages prevalence of self-harm in Iran, stratified by provinces, 1990–2021.

**Figure 11.**
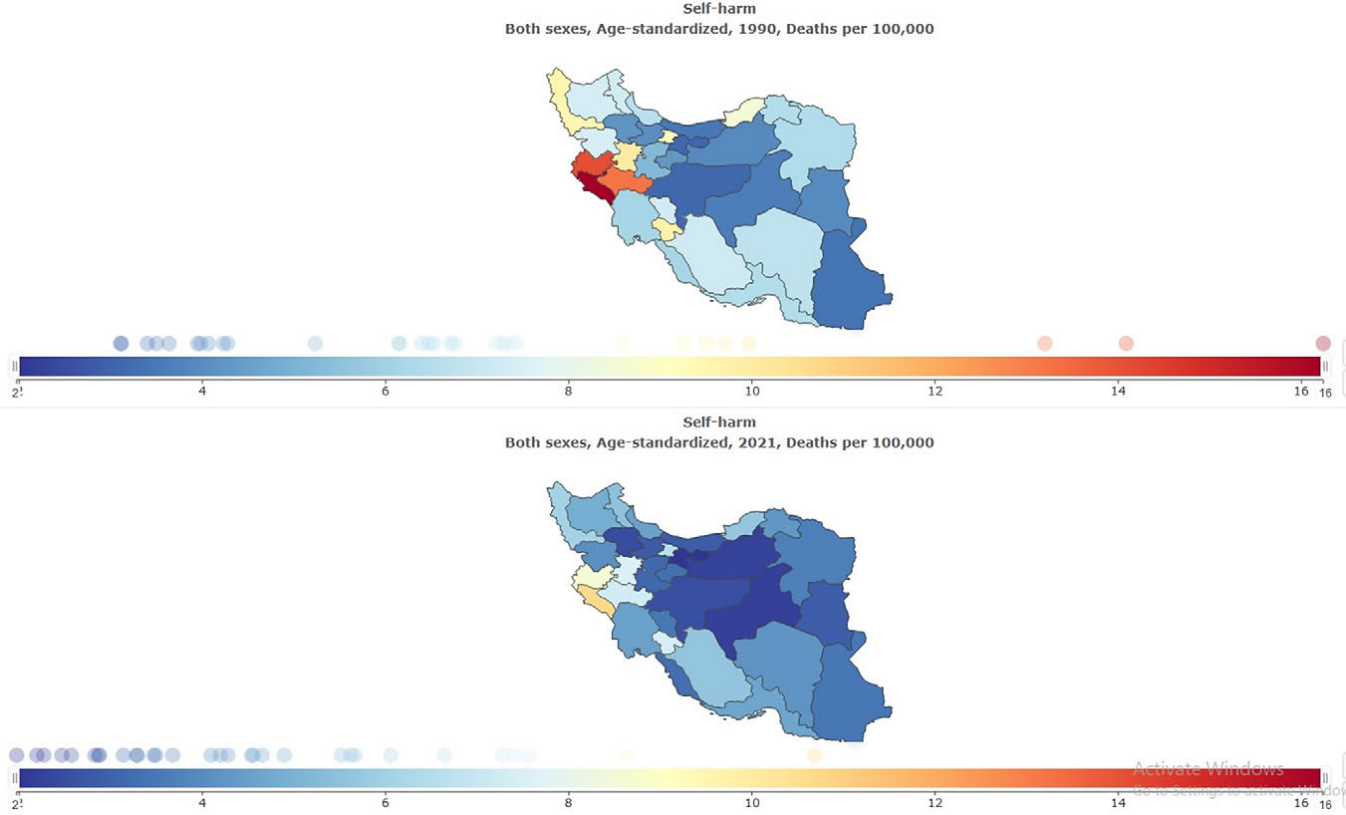
Age-standardized suicide mortality rate (per 100,000) in Iran, stratified by provinces, 1990–2021.

The subnational burden of self-harm and suicide mortality showed that the highest age-standardized prevalence rate per 100,000 in 2021 was in Ilam (188.33 [95% UI: 157.11–223.85]), and the lowest was in Zanjan (106.84 [95% UI: 90.84–127.12]) (Table 1 and Figure 9). The highest self-harm counts were in Tehran (18,736 [95% UI: 15,979–22,234]), and the lowest was in Semnan (945 [95% UI: 799–1,128]) (Table 1 and Figure 10). The highest age-standardized suicide mortality rate per 100,000 was in Ilam (10.68 [95% UI: 4.58–12.93]) (Table 4 and Figure 11).

## Discussion

The present study aimed to investigate the impact of self-harm and suicide mortality across different sex and age groups in Iran from 1990 to 2021. It additionally sought to estimate the prevalence, incidence, disability and mortality rates associated with these issues, analyzing data at both national and subnational levels to provide a comprehensive understanding of their distribution and burden.

Over the past three decades, the patterns of self-harm and suicide mortality in Iran have exhibited a decline. The prevalence, incidence, disability attributed to self-harm and the rates of suicide mortality have shown noticeable reductions compared to figures from 1990. The conclusion of the war against Iran in 1988 marked a period when mental health disorders escalated due to the psychological impacts of war-related trauma. Consequently, the rates of self-harm and suicide mortality in 1990 and the subsequent years were significantly elevated. However, over time, these rates transitioned into a sustained downward trajectory, with notable decreases in the prevalence, incidence and disability associated with self-harm, as well as reductions in suicide mortality observed up to 2021.

Studies indicate that war has a profound impact on the mental health of civilians, leading to a noticeable rise in both the prevalence and incidence of mental disorders (Baingana et al., 2005; Lopez-Ibor et al., 2005; Murthy and Lakshminarayana, 2006). A newly published meta-analysis reveals a notable rise in mental health disorders in regions affected by conflict and war (Lim et al., 2022). Wars often lead to a rise in displaced populations, heightened migration, economic instability and the spread of physical illnesses (Lim et al., 2022). Consequently, this can result in a rise in mental health disorders within the community, potentially contributing to an increase in instances of self-harm and suicide-related fatalities.

During the past decades, Iran has been affected by economic turmoil and international politics (Danaei et al., 2019). During the 1980s, Iran witnessed a marked increase in the birth rate, which led to a significant increase in population growth. This increase in population growth affected all aspects of the health system, so there was an increased demand for health infrastructure, health care and especially mental health-related care. Effective and efficient measures were taken to increase health and health care, but in the area related to mental health care, access to mental health services did not grow proportionately (Farzadfar et al., 2022), and this was one of the factors that affected mental health.

On the other hand, the stigma related to mental disorders is one of the issues that, although it has faded over time, still stands; therefore, the request related to mental health care has always been associated with stigma (Corrigan et al., 2014; Taghva et al., 2017). Another effective factor in mental disorders in Iran during recent decades is due to economic turmoil. Studies have shown that mental disorders, as well as self-harm and suicide, increase as a result of economic crises (World Health Organization. Regional Office for E., 2011; Marazziti et al., 2021). Some of the consequences of economic crises, such as unemployment, poverty and hyperinflation, are associated with an increase in mental disorders, such as depression and suicide, and disrupt mental health (Paul and Moser, 2009; Amiri, 2022a, 2022b).

A part of the economic turmoil was caused by the sanctions imposed on Iran (Kokabisaghi, 2018), which intensified especially since 2011. Although the factors affecting mental disorders, especially self-harm and suicide mortality, have had cumulative effects in Iran over the past decades, unlike depression and anxiety disorders, the prevalence of self-harm and suicide mortality is low in Iran, and it is lower than the world average and many countries with a high level of social welfare. The dominant religion of Iran is Islam, and in most Islamic countries, the suicide rate is low (Pritchard and Amanullah, 2007; Mirhashemi et al., 2016; Lew et al., 2022). This could be partly due to the role of religion. In Islam, suicide and self-harm are prohibited, and the sanctity of life is emphasized in Islamic teachings (Ali, 2000). Of course, less access to mental health care alone is not the determining factor. There is a stigma attached to suicide for families (Wyllie et al., 2025). The stigma associated with suicide is important in Iranian culture (Masoomi et al., 2022). One significant obstacle to accessing mental health services in Iran is the social stigma surrounding psychiatric issues (Taghva et al., 2017). Research conducted in Iran indicates that women often attempt to conceal their suicide attempts due to the fear of being stigmatized by society (Azizpour et al., 2018), and the burden of this stigma causes many individuals and families to not seek mental health care despite the need for mental health professionals and mental health care providers (Tay et al., 2018). Suicide is heavily stigmatized in predominantly Muslim countries like Iran, and similarly, other religious communities are not immune to this issue (Shoib et al., 2022).

The differences between sexes in self-harm and suicide mortality have consistently drawn the attention of researchers. Studies exploring these disparities reveal that suicide mortality is notably higher among males compared to females. Data published by the WHO further supports this finding, highlighting that, on a global scale, the age-standardized suicide rate for males is 2.3 times greater than that of females (World Health Organization, 2021). Major depressive disorder constitutes a significant factor contributing to suicide. While its prevalence is observed to be twice as high among females compared to males, the likelihood of suicide among females is markedly lower, amounting to only one-fourth of the probability seen in males (Murphy, 1998). The higher incidence of suicide among men has been attributed to several factors, including contrasting behavioral and emotional tendencies between genders. Men often prioritize independence and decisiveness, perceiving the admission of needing assistance as a sign of weakness, which leads them to avoid seeking help. On the other hand, women tend to emphasize interdependence and are more likely to seek support from friends and accept help readily, contributing to differing responses in times of emotional distress (Murphy, 1998).

This study represents the first comprehensive endeavor to analyze trends in self-harm and suicide in Iran since 1990, offering gender- and age-specific estimates. However, it is subject to certain limitations extensively discussed within the framework of the GBD study. Challenges include issues related to the quality and collection of primary data, as well as inconsistencies in data availability. Moreover, the risk factors assessed are constrained by the exclusion of various potentially significant risk factors and covariates. The impact of the coronavirus disease 2019 pandemic further complicates the interpretation and analysis of findings. A more detailed discussion on the methodological constraints inherent in the GBD study is available in other sources (Brauer et al., 2024; Ferrari et al., 2024). GBD estimates are often derived from modeled data, particularly in contexts where the quality of surveillance systems is insufficient or constrained

## Health implications

The gathered data highlights the trends of suicide and suicide mortality in Iran over the past three decades. Despite notable advancements in physical health within the Iranian healthcare system during this period, demographic shifts and lifestyle changes have exposed gaps in addressing mental health. Efforts to improve awareness around mental health and ensure broader access to mental health services have remained insufficient. To address these challenges effectively, it is crucial to prioritize mental health literacy and expand access to care as key components in health policy development.

## Conclusion

The present study showed that despite demographic changes, economic turmoil and war, self-harm and suicide mortality have had a decreasing trend in Iran. Suicide mortality in males was higher than in females, self-harm was more common in females in past decades, and in recent years, it was lower than in males.

## Data Availability

The data sources of this study were taken from GBD 2021, which is publicly available. In this way, the data can be accessed through the links below: https://vizhub.healthdata.org/gbd-results/ https://vizhub.healthdata.org/gbd-compare/

## Abbreviations

DALYs: disability-adjusted life years
GBD: global burden of disease, encapsulating a comprehensive assessment of health losses due to diseases and injuries worldwide
ICD: International Classification of Diseases, providing a standard framework for categorizing health conditions
SEV: summary exposure value, quantifying exposure levels to specific risk factors
YLDs: years lived with a disability
YLLs: years of life lost due to premature death

## References

[r1] Ali AY (2000) *The holy Qur’an*: Wordsworth Editions.

[r2] Amiri S (2022a) Unemployment and suicide mortality, suicide attempts, and suicide ideation: A meta-analysis. International Journal of Mental Health 51(4), 294–318.

[r3] Amiri S (2022b) Unemployment associated with major depression disorder and depressive symptoms: A systematic review and meta-analysis. International Journal of Occupational Safety and Ergonomics 28(4), 2080–2092. 34259616 10.1080/10803548.2021.1954793

[r4] Azizpour M, Taghizadeh Z, Mohammadi N and Vedadhir A (2018) Fear of stigma: The lived experiences of Iranian women after suicide attempt. Perspectives in Psychiatric Care 54(2), 293–299. 10.1111/ppc.12237. 29165826

[r5] Baingana FK, Bannon I and Thomas R (2005) Mental Health and Conflicts: Conceptual Framework and Approaches. World Bank Washington. https://documents.worldbank.org/en/publication/documents-reports/documentdetail/829381468320662693

[r6] Bank TW (2021) GDP per capita (current US$)—Iran, Islamic Rep. Available at https://data.worldbank.org/indicator/NY.GDP.PCAP.CD?end=2018&locations=IR&start=1960

[r7] Bhattacharjee NV, Schumacher AE, Aali A, Abate YH, Abbasgholizadeh R, Abbasian M, … Vollset SE (2024) Global fertility in 204 countries and territories, 1950–2021, with forecasts to 2100: A comprehensive demographic analysis for the Global Burden of Disease Study 2021. The Lancet 403(10440), 2057–2099. 10.1016/S0140-6736(24)00550-6. PMC11122687 38521087

[r8] Brauer M, Roth GA, Aravkin AY, Zheng P, Abate KH, Abate YH, … Gakidou E (2024) Global burden and strength of evidence for 88 risk factors in 204 countries and 811 subnational locations, 1990–2021: A systematic analysis for the global burden of disease study 2021. The Lancet 403(10440), 2162–2203. 10.1016/S0140-6736(24)00933-4. PMC11120204 38762324

[r9] Corrigan PW, Druss BG and Perlick DA (2014) The impact of mental illness stigma on seeking and participating in mental health care. Psychological Science in the Public Interest 15(2), 37–70. 10.1177/1529100614531398. 26171956

[r10] Danaei G, Farzadfar F, Kelishadi R, Rashidian A, Rouhani OM, Ahmadnia S, … Arhami M (2019) Iran in transition. The Lancet 393(10184), 1984–2005. 10.1016/S0140-6736(18)33197-0 31043324

[r11] Farzadfar F, Naghavi M, Sepanlou SG, Saeedi Moghaddam S, Dangel WJ, Davis Weaver N, … Larijani B (2022) Health system performance in Iran: A systematic analysis for the global burden of disease study 2019. The Lancet 399(10335), 1625–1645. 10.1016/S0140-6736(24)00757-8. PMC9023870 35397236

[r12] Ferrari AJ, Santomauro DF, Aali A, Abate YH, Abbafati C, Abbastabar H, … Murray CJL (2024) Global incidence, prevalence, years lived with disability (YLDs), disability-adjusted life-years (DALYs), and healthy life expectancy (HALE) for 371 diseases and injuries in 204 countries and territories and 811 subnational locations, 1990–2021: A systematic analysis for the global burden of disease study 2021. The Lancet 403(10440), 2133–2161. 10.1016/S0140-6736(24)00757-8. PMC11122111 38642570

[r13] GBD 2021 Causes of Death Collaborators (2024) Global burden of 288 causes of death and life expectancy decomposition in 204 countries and territories and 811 subnational locations, 1990-2021: A systematic analysis for the global burden of disease study 2021. Lancet 403(10440), 2100–2132. 10.1016/s0140-6736(24)00367-2. 38582094 PMC11126520

[r14] Global Health Metrics G. (2024) Self-harm—Level 3 cause. Available at https://www.healthdata.org/research-analysis/diseases-injuries-risks/factsheets/2021-self-harm-level-3-disease

[r15] Knipe D, Padmanathan P, Newton-Howes G, Chan LF and Kapur N (2022) Suicide and self-harm. The Lancet 399(10338), 1903–1916. 10.1016/S0140-6736(22)00173-8. 35512727

[r16] Kokabisaghi F (2018) Assessment of the effects of economic sanctions on Iranians’ right to health by using human rights impact assessment tool: A systematic review. International Journal of Health Policy and Management 7(5), 374–393. 10.15171/ijhpm.2017.147. 29764102 PMC5953521

[r17] Lew B, Lester D, Kõlves K, Yip PSF, Chen YY, Chen WS, … Ibrahim N (2022) An analysis of age-standardized suicide rates in Muslim-majority countries in 2000-2019. BMC Public Health 22(1), 882. 10.1186/s12889-022-13101-3. 35509027 PMC9066769

[r18] Lim ICZY, Tam WWS, Chudzicka-Czupała A, McIntyre RS, Teopiz KM, Ho RC and Ho CSH (2022) Prevalence of depression, anxiety and post-traumatic stress in war- and conflict-afflicted areas: A meta-analysis. Frontiers in Psychiatry 13, 978703. 10.3389/fpsyt.2022.978703. PMID: 36186881; PMCID: PMC9524230. 36186881 PMC9524230

[r19] Lopez-Ibor JJ, Christodoulou G, Maj M, Sartorius N and Okasha A (2005) Disasters and Mental Health. Wiley Online Library.

[r20] Marazziti D, Avella MT, Mucci N, Della Vecchia A, Ivaldi T, Palermo S and Mucci F (2021) Impact of economic crisis on mental health: A 10-year challenge. CNS Spectrums 26(1), 7–13. 10.1017/S1092852920000140. 32252843

[r21] Masoomi M, Hosseinikolbadi S, Saeed F, Sharifi V, Jalali Nadoushan AH and Shoib S (2022) Stigma as a barrier to suicide prevention efforts in Iran. Frontiers in Public Health 10, 1026451. 10.3389/fpubh.2022.1026451. 36699938 PMC9868841

[r22] Mirhashemi S, Motamedi MHK, Mirhashemi AH, Taghipour H and Danial Z (2016) Suicide in Iran. The Lancet 387(10013), 29. 10.1016/S0140-6736(15)01296-9. 26766345

[r23] Moradinazar M, Mirzaei P, Moradivafa S, Saeedi M, Basiri M and Shakiba M (2022) Epidemiological status of depressive disorders in the Middle East and North Africa from 1990 to 2019. Health Promotion Perspective 12(3), 301–309. 10.34172/hpp.2022.39. PMC9808901 36686044

[r24] Murphy GE (1998) Why women are less likely than men to commit suicide. Comprehensive Psychiatry 39(4), 165–175. 10.1016/s0010-440x(98)90057-8. 9675500

[r25] Murthy RS and Lakshminarayana R (2006) Mental health consequences of war: A brief review of research findings. World Psychiatry 5(1), 25–30. 16757987 PMC1472271

[r26] Nazarzadeh M, Bidel Z, Ayubi E, Asadollahi K, Carson KV and Sayehmiri K (2013) Determination of the social related factors of suicide in Iran: A systematic review and meta-analysis. BMC Public Health 13, 4. 10.1186/1471-2458-13-4. 23289631 PMC3627903

[r27] Paul KI and Moser K (2009) Unemployment impairs mental health: Meta-analyses. Journal of Vocational Behavior 74(3), 264–282.

[r28] Pritchard C and Amanullah S (2007) An analysis of suicide and undetermined deaths in 17 predominantly Islamic countries contrasted with the UK. Psychological Medicine 37(3), 421–430. 10.1017/s0033291706009159. 17176500

[r29] Sharifi V, Amin-Esmaeili M, Hajebi A, Motevalian A, Radgoodarzi R, Hefazi M and Rahimi-Movaghar A (2015) Twelve-month prevalence and correlates of psychiatric disorders in Iran: The Iranian mental health survey, 2011. Archives of Iranian Medicine 18(2), 76–84. 25644794

[r30] Shoib S, Armiya’u AY u, Nahidi M, Arif N and Saeed F (2022) Suicide in Muslim world and way forward. Health science reports 5(4), e665. 35702511 10.1002/hsr2.665 PMC9178353

[r31] Stevens GA, Alkema L, Black RE, Boerma JT, Collins GS, Ezzati M, … Horton R (2016) Guidelines for accurate and transparent health estimates reporting: The GATHER statement. The Lancet 388(10062), e19–e23. 10.1016/S0140-6736(16)30388-9 27371184

[r32] Taghva A, Farsi Z, Javanmard Y, Atashi A, Hajebi A and Khademi M (2017) Stigma barriers of mental health in Iran: A qualitative study by stakeholders of mental health. Iranian Journal of Psychiatry 12(3), 163–171. 29062367 PMC5640577

[r33] Tay S, Alcock K and Scior K (2018) Mental health problems among clinical psychologists: Stigma and its impact on disclosure and help-seeking. Journal of Clinical Psychology 74(9), 1545–1555. 10.1002/jclp.22614. 29573359

[r34] World Health Organization (2019) Suicide and Self-Harm. Cairo: Regional Office for the Eastern, M

[r35] World Health Organization. (2021). Suicide worldwide in 2019: global health estimates.

[r36] World Health Organization (2023) Suicide. Regional Office for the Eastern, M

[r37] World Health Organization (2014). *Preventing suicide: A global imperative*: World Health Organization.

[r38] World Health Organization. Regional Office for, E. (2011). *Impact of economic crises on mental health.* Available at Copenhagen: https://iris.who.int/handle/10665/370872

[r39] Wyllie JM, Robb KA, Sandford D, Etherson ME, Belkadi N and O’Connor RC (2025) Suicide-related stigma and its relationship with help-seeking, mental health, suicidality and grief: Scoping review. BJPsych Open 11(2), e60. 10.1192/bjo.2024.857. 40116563 PMC12001961

